# Modulation of blood T cell polyfunctionality and HVEM/BTLA expression are critical determinants of clinical outcome in anti-PD1-treated metastatic melanoma patients

**DOI:** 10.1080/2162402X.2024.2372118

**Published:** 2024-06-26

**Authors:** Stéphane Dalle, Estelle Verronese, Axelle N’Kodia, Christine Bardin, Céline Rodriguez, Thibault Andrieu, Anais Eberhardt, Gabriel Chemin, Uzma Hasan, Myrtille Le-Bouar, Julie Caramel, Mona Amini-Adle, Nathalie Bendriss-Vermare, Bertrand Dubois, Christophe Caux, Christine Ménétrier-Caux

**Affiliations:** a Department of Dermatology, Centre Hospitalier Lyon Sud, Hospices Civils de Lyon, Lyon 1 University, Lyon, France; b Cancer Research Center of Lyon, INSERM 1052 - CNRS 5286, Centre Léon Bérard, Université de Lyon, Université Claude Bernard Lyon 1, Lyon, France; c Lyon Immunotherapy for Cancer Laboratory (LICL), Centre Léon Bérard, Lyon, France

**Keywords:** Advanced melanoma, anti-PD1, biomarkers of response, blood immuno-monitoring, HVEM, T cell polyfunctionality

## Abstract

The need for reliable biomarkers to predict clinical benefit from anti-PD1 treatment in metastatic melanoma (MM) patients remains unmet. Several parameters have been considered in the tumor environment or the blood, but none has yet achieved sufficient accuracy for routine clinical practice. Whole blood samples from MM patients receiving second-line anti-PD1 treatment (NCT02626065), collected longitudinally, were analyzed by flow cytometry to assess the immune cell subsets absolute numbers, the expression of immune checkpoints or ligands on T cells and the functionality of innate immune cells and T cells. Clinical response was assessed according to Progression-Free Survival (PFS) status at one-year following initiation of anti-PD1 (responders: PFS > 1 year; non-responders: PFS ≤ 1 year). At baseline, several phenotypic and functional alterations in blood immune cells were observed in MM patients compared to healthy donors, but only the proportion of polyfunctional memory CD4+ T cells was associated with response to anti-PD1. Under treatment, a decreased frequency of HVEM on CD4+ and CD8+ T cells after 3 months of treatment identified responding patients, whereas its receptor BTLA was not modulated. Both reduced proportion of CD69-expressing CD4+ and CD8+ T cells and increased number of polyfunctional blood memory T cells after 3 months of treatment were associated with response to anti-PD1. Of upmost importance, the combination of changes of all these markers accurately discriminated between responding and non-responding patients. These results suggest that drugs targeting HVEM/BTLA pathway may be of interest to improve anti-PD1 efficacy.

## Introduction

Over the past decade, medical authorities have approved the use of antibodies against the immune checkpoint (ICP) PD1 or its ligand PDL1 as a treatment for metastatic melanoma (MM), based on their strong therapeutic efficacy and ability to extend long-term survival in some patients.^1,2^ Anti-PD1/L1 therapy aims to block the interaction between PD1 on tumor-reactive T cells and PD1 ligands (PDL1 and PDL2) expressed on various cell types, including tumor cells and leukocytes (for review^3^). However, despite improved clinical responses and long-term survival benefits for MM patients, approximately two-thirds of treated patients do not experience clinical benefit.^4^ In addition, ICP blockade is associated with frequent adverse events, which are sometime long-lasting and irreversible.^5^ Identifying reliable criteria for distinguishing responding (R) from non-responding (NR) patients prior to, or early during, treatment is crucial to prospectively identify patients for whom the benefit/risk ratio is the highest to improve melanoma patient care as opposed to those with the lowest benefit/risk ratio for which therapeutic abstention or early termination could mitigate adverse events and allow to evaluate alternative treatments.

In MM, several tumor biomarkers have been proposed to predict the response or resistance to anti-PD1 treatment. Tumor-cell intrinsic features such as the expression of PDL1,^6,7^ the reduced load of tumor neo-antigens^8^ or the defects in antigen presentation and IFN-γ pathways^9^ have been associated with resistance to anti-PD1. On the contrary, immune cell features such as high frequency of infiltrating CD8+ T cells before or early during treatment,^6,10^ the immune spatial organization into tertiary lymphoid structures,^11,12^ or the detection of specific immune niches^13–15^ have been reported as markers of anti-PD1 efficacy. However, none of these individual immune parameters or their combination is currently used in clinical practice to predict anti-PD1/L1 treatment response.

The analysis of blood-based biomarkers before and during treatment represents a noninvasive way to monitor immune response to anti-PD1. Over the past five years, several studies in patients with MM and non-small cell lung cancer (NSCLC) found that low levels of soluble PD1 and PDL1 in the plasma before or during treatment have been associated with good response to treatment in NSCLC,^13–15^ but this does not apply to MM.^16^ Most of pre- and on-treatment blood-based biomarkers associated with the efficacy of anti-PD1 in patients with different types of tumors (NSCLC, renal cell carcinoma, MM, MSI-high gastric cancer) focused on routine blood parameters (such as neutrophil, eosinophil, or lymphocyte counts or ratios) or changes in blood immune cells phenotype. Yet, to date, none of these blood immune parameters is used in clinical practice to predict the outcome of anti-PD1 treatment and adapt the therapeutic regimen.^17,18^

Importantly, there is so far limited information regarding immune blood cell functionality and response to anti-PD1. In this respect, we developed a whole-blood assay to monitor the functional competence of innate immune cells (NK cells, dendritic cells (DC), monocyte subsets) and T cells by measuring, by multi-parametric flow-cytometry (FC), their ability to produce specific cytokines after short-term *in vitro* stimulation with TLR ligands or polyclonal activation, respectively.^19^ Using these assays, we revealed in BC patients several functional alterations of circulating innate and adaptive immune cells that were exacerbated when metastases were present.^19^ These results reinforce the importance of monitoring blood innate and adaptive immunity to identify dysfunctions associated with tumor progression, patient survival and/or response to ICP blockers.

Here, we conducted such comprehensive analysis by FC analysis on fresh whole blood samples in MM patients prior to and upon anti-PD1 second line therapy, compared to healthy donors (HD) with a particular focus on T cells phenotype and memory T cells function known as one of the targets of anti-PD1 treatment (for review).^20^

At baseline, we highlighted several defaults in phenotypic and functional characteristics of blood immune cells in MM, but only proportion of polyfunctional CD4+ memory T cells was associated with response to anti-PD1. Upon anti-PD1, we highlighted a significant decrease in herpesvirus entry mediator (HVEM) expression on CD4+ and CD8+ T cells in responders without modulation of its receptor B- and T-lymphocyte attenuator (BTLA). We also identified increased numbers and polyfunctional capacities of memory CD4+ and CD8+ T cells in patients who responded to anti-PD1. Of importance, the combination of changes of all these markers during the first three months of anti-PD1 treatment accurately discriminated between R and NR patients.

## Material and methods

### Patients

PAIR cohort (NCT02626065) is a single center cohort of MM patients (*n* = 29) treated with nivolumab (BMS, 3 mg/kg, i.v., every 3 weeks) as second line following treatment with targeted therapy (BRAFi ± MEKi) for patients with BRAF^V600E^ mutation (*n* = 18) or anti CTLA-4 (ipilimumab) for patients with wild-type BRAF (*n* = 11). The protocol was approved by a regional review board (Comite de Protection des Personnes Sud-Est IV, France, number 2014-038-2). The generation of a PBMC collection in the PAIR cohort was approved by French National Agency for the Safety of Medicinal Products and Health Products ANSM (ref 141–391-A12). Patients were included and followed between April 2015 and July 2017. All patients enrolled in this trial provided a signed informed consent in accordance with the Declaration of Helsinki. Tumor assessments were made every 12 weeks from the time of administration of the first anti-PD1 dose, by performing evaluation of the tumor extension by CTAP scan reviewed according to RECIST1.1 criteria. Patients’ clinical response to anti-PD1 therapy was determined according to the Progression-Free Survival (PFS) status at one-year following initiation of anti-PD1 treatment (R patients: PFS >1 year; NR: PFS ≤1 year). Associated clinical data were collected according to the protocol.

### Whole blood collection

For each patient, 12 mL whole blood was collected at different time points (inclusion (prior anti-PD1), Week (W)2, W12 and W54) using EDTA- or Heparin-coated tubes for phenotypic and functional analyses respectively. Whole blood samples from HD controls were obtained from the French blood agency (Etablissement Français du sang, Lyon France, convention 16–093).

### Whole blood immune cell subsets quantification and analysis of ICP and ICP ligands on T cells

Characterization of innate and adaptive immune cell subsets was performed by FC using specific surface marker combinations (see Supplementary Table S1a-b). Briefly, 1 million whole blood cells were incubated, after erythrocytes lysis (BD Pharmlyse®, BD Biosciences, 10 min, room temperature), with a viability marker (Live and dead NIR, BD Biosciences) for 15 min at room temperature. Cells were then washed and stained with surface antibodies to detect specific cell subsets at 4°C for 30 min. After washes, cells were fixed in formaldehyde 4%. For regulatory T cells (Treg) characterization, cells were treated using the FOXP3 Fixation and Permeabilization kit (Life Technologies) and then stained intracellularly with anti-FOXP3 antibody (Supplementary Table S1c). In parallel, ICP (PD1, TIGIT, BTLA, CD69, CD73, CD137) and ICP ligands (ICP-L) (PDL1, HVEM) expression on total CD4+ and CD8+ T cells was analyzed by staining with surface specific antibodies (Supplementary Table S1d) at 4°C for 30 min. After washes, cells were fixed in formaldehyde 4%. All FC acquisitions were done on a FACS-CANTO-2 (BD Biosciences), and data were processed with DIVA software (BD Biosciences) (Supplementary Figure S1).

### Functional characterization of innate and adaptive immune cells

1 million whole blood cells were subjected to 5 h reactivation step in complete RPMI medium containing human sAB (EFS) with either Toll Like Receptor (TLR) Ligand (InVivogen: R848 (TLR7/8), 5 µg/mL or poly I:C (TLR3), 30 µg/mL) or phorbol myristate acetate (PMA) (50 ng/mL)/ionomycin (1 µg/mL) (Sigma-Aldrich) in the presence of the secretion inhibitor GolgiPLug (BD Biosciences) to analyze by FC the functional capacity of respectively innate immune cells (DC subsets, monocytes and NK cells) and T cell subsets. At the end of activation, erythrocytes were lysed and harvested cells were stained with membrane markers panel for “innate immune cell” (Supplementary Figure S2a) or “T cell” activation (Supplementary Figure S2b). Then cells were fixed, permeabilized and stained for dedicated intracellular cytokines. After resuspension in 600 µL of staining buffer (PBS containing 2% FCS (Eurobio) and 1 mM EDTA (Sigma-Aldrich)), all events were acquired on a LSRII Fortessa cell analyzer (BD Biosciences). Data were processed in FlowJo software v9.6.4 (TreeStar, Inc.) and cytokine secretion by different cell subsets defined by the gating strategy as previously described^19^ was evaluated by creation of Boolean gates. The polyfunctionality of memory CD4+ and CD8+ T cells was assessed by the cumulation of Boolean gates containing cells co-producing IFN-γ and TNF-α.

### Statistical analyses

The statistical differences between patients and HD and between R and NR patients at time of inclusion were assessed using a Mann-Whitney U test. The statistical differences during treatment on both R and NR patients was assessed with a two-way ANOVA parametric analysis with a Tukey’s correction for multiple comparisons. The statistical differences between R and NR patients for evolution of biological parameters during treatment (difference between W12 (or W2) and inclusion) were assessed using a Mann-Whitney U test. Results were considered as statistically significant when *p* value was < .05 (*: *p* value < .05; **: *p* value < .01; ***: *p* value < .001; ****: *p* value < 10^−4^).

For principal component analysis (PCA), we integrated all the parameters significantly associated with response to treatment on CD4+ and/or CD8+ T cells. As HVEM modulation (W12- inclusion) on CD4+ and CD8+ T cells did not present a normal distribution and presented negative values, raw data were first translated into positive values by adding a constant value (1- min(x)) to each raw data and then transformed into Log10 to obtain normalized distribution. All these parameters were integrated in the model to decipher their capacity to discriminate R from NR patients.

### ROC curve generation and AUC calculation

The dataset consisted of immunological markers from patients with their binary response to treatment (R or NR). A random forest (RF) machine learning model was developed to predict patient response on R (v4.3.3). ROCR package was used to compute the sensitivity (true positive rate) and the 1-specificity (false positive rate). Individual ROC curves were generated for each immunological marker and the combination of markers from RF model predictions alongside the collective analysis. The Area Under the Curve (AUC) was calculated using pROC package.

## Results

### Description of the cohort and clinical evolution

At baseline, the median age was 61 [33–92] years, 65% were male and 35% were female, 24% had an elevated lactate dehydrogenase (LDH, 7/29). 62% of patients had a BRAF V600 mutation, and 7% had a NRAS mutation. 83% of patients were ECOG performance status 0 or 1. The median Breslow at diagnosis was 3.6 mm [0.7–18]. Ulceration of the primary lesion was observed in 45% of patients. There were 24% patients with brain metastases at the time of anti-PD1 treatment onset. There was no significant difference in clinical patient characteristics according to the response to anti-PD1 treatment, except for a significantly higher proportion of patient with Non-Determined (ND) status for ulceration of the primary lesion in NR (*p* = .047) (Supplementary Table S2).

### MM patients harbor phenotypic and functional alterations in blood immune cells prior to anti-PD1 treatment

**Figure 1.**
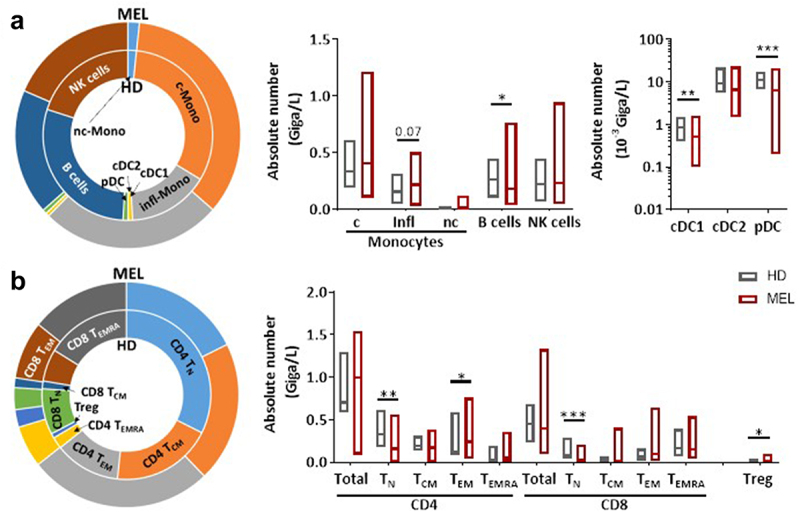
Melanoma patients present several alterations in peripheral immune cells compared to HD.

Counts of white blood cells, neutrophils, T cells, NK cells and monocyte subsets did not significantly differ between MM patients (*n* = 29) prior to anti-PD1 treatment and HD controls (*n* = 16) (Figure 1(a)). However, we observed a significant decrease in the number of B cells and all DC subsets in MM patients (Figure 1(a), Supplementary Figure S1a). In addition, while total number of CD4+ and CD8+ T cells were similar between MM patients and HD, we found a reduction in the absolute number of CD4+ and CD8+ naive T cells (T_N_) and an increase of CD4+ effector memory T cells (T_EM_) and T_EMRA_ in MM patients (Figure 1(b), Supplementary Figure S1a).

Phenotypic analysis of whole blood from MM patients (*n* = 29) before treatment and compared to HD (*n* = 16) based on FC panels presented in Supplementary Table 1A-C and Supplementary Fig.1A. Analysis of absolute number of APCs (DC and monocyte subsets, B cells) and NK cells (a). Analysis of CD4+ and CD8+ T cell subsets according to their differentiation stage (T_N_, T_CM_, T_EM_, T_EMRA_) and Treg (b). The median of each group was presented. Patients and HD were compared using Mann-Whitney U test.

**Figure 2.**
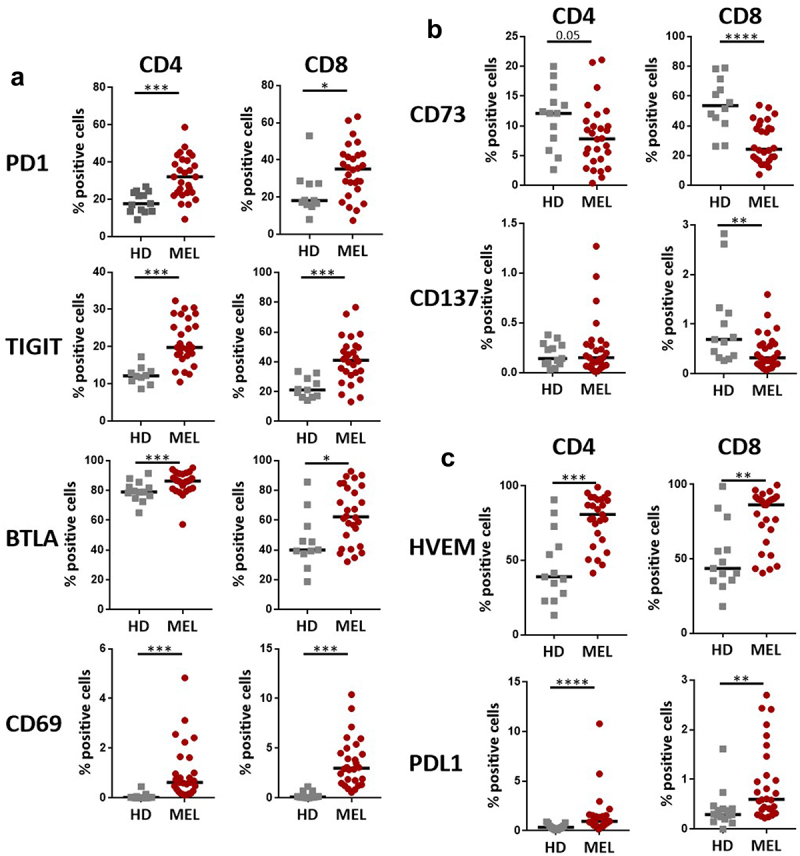
Melanoma patients present increased expression of different ICP on T cells subsets.

Analysis of the expression of ICPs on total CD4+ and CD8+ T cells revealed an increased proportion of T cells expressing PD1 in MM patients (CD4+ T cells: median = 32.13%; CD8+ T cells: median = 35.1%) compared to HD (CD4+ T cells: median = 17.68%; CD8+ T cells: median = 18.2%) (Figure 2(a), Supplementary Figure S1b). When compared to HD, MM patients also showed an upregulation of TIGIT, BTLA and CD69, and a downregulation of CD73 on both total CD4+ and CD8+ T cells (Figure 2(a,b)). We also analyzed the expression of the ICP-L HVEM and PDL1. Even if the proportion of cells expressing PDL1 remained very low in both total CD4+ and CD8+ T cells, it was significantly higher in MM patients compared to HD (Figure 2(c)). Of interest, we noticed a higher proportion of total CD4+ and CD8+ T cells expressing HVEM in MM patients compared to HD (Figure 2(c)).

The expression of ICP (PD1, TIGIT, BTLA, CD69, CD73, CD137) and ICP-L (HVEM, PDL1) was analyzed by flow cytometry panels (see Supplementary Table S1d and Supplementary Figure1b for description) on total blood CD4+ and CD8+ T cells. The median of each group was presented. Patients and HD were compared using Mann-Whitney U test.

**Figure 3.**
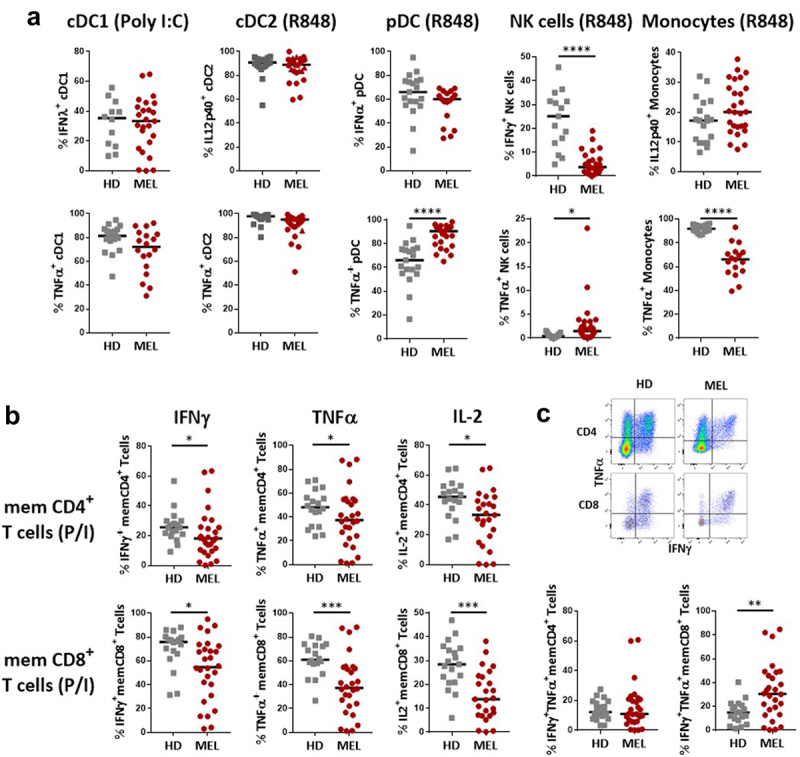
Melanoma patients blood immune cells present reduced capacity to produce cytokines compared to HD.

We then evaluated the functionality of innate and adaptive immune cells in the blood by measuring cytokine production by FC after *ex vivo* short-term whole blood stimulation as previously described.^19^ For innate immune cell stimulation, ligands of TLR7/8 or TLR3 were used to activate conventional DC2 (cDC2), plasmacytoid DC (pDC) and monocytes vs cDC1, respectively. In MM patients, an increased proportion of TNF-α-expressing pDC was observed, that contrasted with the decreased proportion of TNF-α-expressing monocytes (Supplementary Figure S2a, Figure 3(a)). The production of IFN-α by pDCs and of IFN-λ by cDC1 remained unchanged (Supplementary Figure S2a, Figure 3(a)). We also observed a sharp decrease in the proportion of NK cells producing IFN-γ in MM patients (Supplementary Figure S2a, Figure 3(a)).

Whole blood cells collected on heparin were subjected to short-term stimulation with either TLR7/8 ligand (R848) or PMA/ionomycin in the presence of secretion inhibitor (Golgi Plug) to investigate respectively their capacity to produce cytokines analyzed by FC (see **Supplementary Figure S2** for panel description). (a) Cytokines production by innate immune cells after R848 reactivation. (b) Cytokines production by T cell subsets after PMA/ionomycin reactivation. (c) Capacity of memory CD4+ and CD8+ T cells to co-secrete IFN-γ and TNF-α. The median of each group was presented. Patients and HD were compared using Mann-Whitney U test.

A similar analysis performed on T cells following *ex vivo* reactivation with P/I revealed that the frequency of cytokine (IFN-γ, TNF-α, IL-2) producing memory (CD45RA^neg^) effector (_TEM_) and central memory (T_CM_) CD4+ or CD8+ T cells was highly heterogeneous in MM patients compared to HD (Supplementary Figure S2b, Figure 3(b)). Furthermore, while the ability of memory T cells to produce TNF-α and IL-2, and to a lesser extend IFN-γ, was altered in MM patients, these effects were more pronounced in CD8+ cells compared to CD4+ T cells (Supplementary Figure S2a, Figure 3(b)). Memory T cell polyfunctionality was assessed by quantifying the proportion of cells co-expressing IFN-γ and TNF-α in response to activation. At baseline, while polyfunctionality of memory CD4+ T cells was not merely different between MM patients and HD, that of memory CD8+ T cells appeared significantly higher in MM patients (Figure 3(c)).

Overall, our findings demonstrate for the first time that MM patients exhibit significant peripheral immune dysfunction, affecting T cells, B cells, DCs, NK cells, and memory CD8+ T cell function, which are likely related to the severity of the disease.

### Response to anti-PD1 treatment is not associated with any baseline phenotypic and functional blood immune cell features except CD4+ memory T cell polyfunctionality

We did not observe any correlation between the absolute number of immune cell populations (Supplementary Table 3a) nor their expression of ICPs or ICP-Ls (Supplementary Table S3b) at baseline and response to anti-PD1, assessed by the absence of progression at 12 months. Additionally, the PERLS score (CD8+PD1+/CD4+PD1+ ratio)^21^ and neutrophil-to-lymphocyte ratio (NLR), previously identified as associated, respectively, with anti-PD1 response in NSCLC^21^ and MM^22^ did not distinguish R patients from NR patients at the time of inclusion (Supplementary Table 3b). While we observed no significant difference in the functionality of innate immune cells between R and NR patients (Supplementary Table S3c), we unexpectedly noticed, at baseline, a reduced capacity to produce IFN-γ in memory CD4+ T cells (*p* = .041) and, to a lesser extent, in memory CD8+ T cells (*p* = .064) from R patients compared to NR patients (Supplementary Table S1c). We also highlighted a reduced polyfunctionality of memory CD4+ T cells in R patients (*p* = .036) (Supplementary Table S3c).

### HVEM expression on T cells is downregulated upon treatment in responding patients

**Figure 4.**
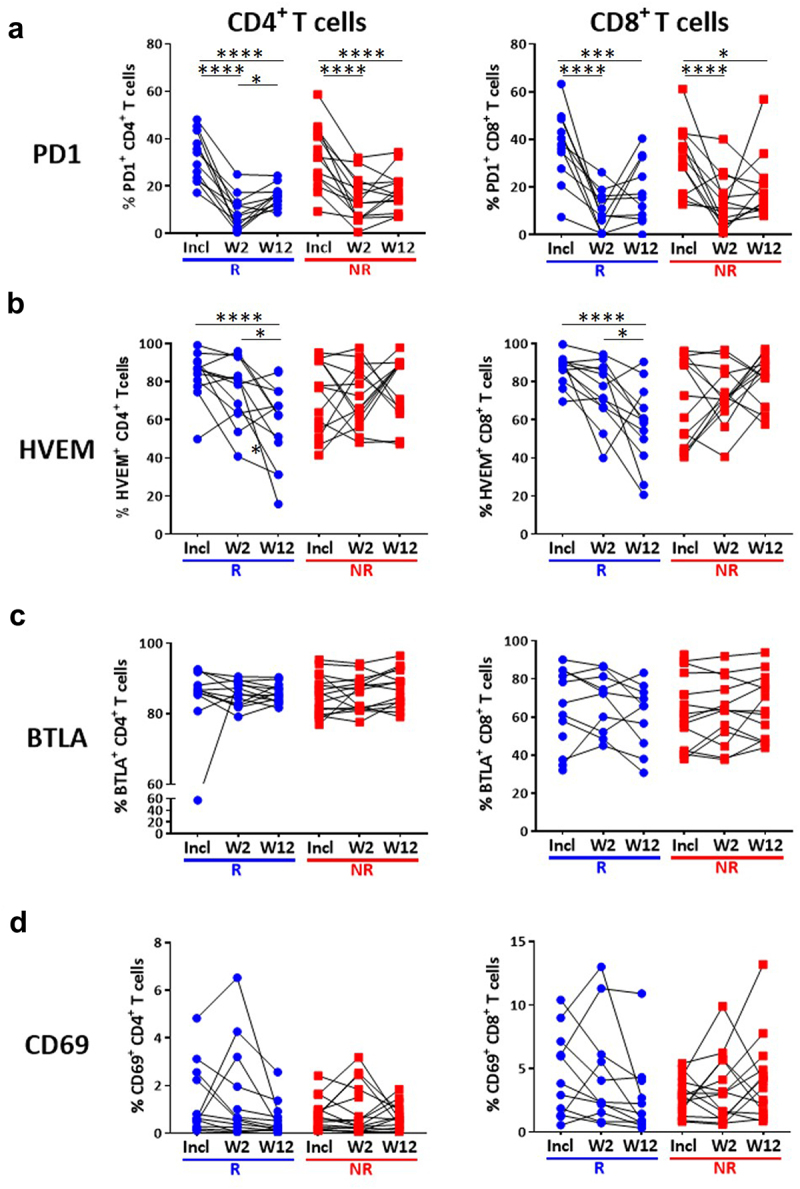
Decreased HVEM expression on T cells in patients responding to treatment.

**Figure 5.**
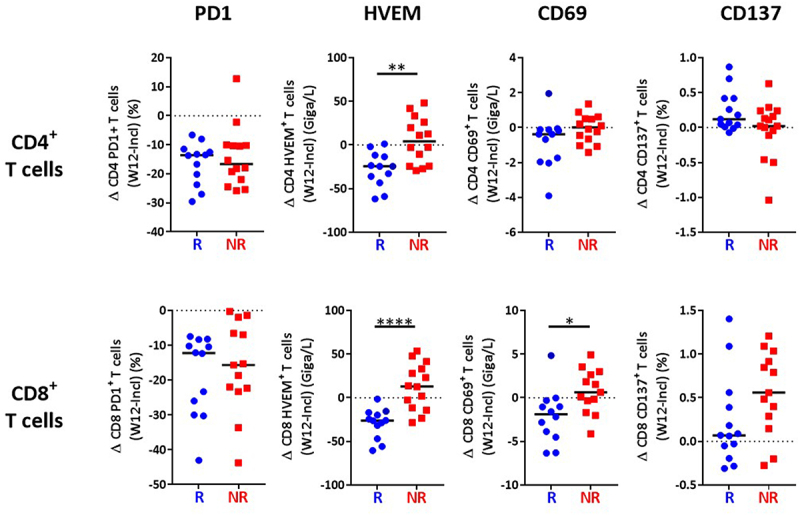
Decreased HVEM expression on T cells after 3 months of treatment in responding patients.

We then investigated the evolution of immune parameters during treatment and their link with the response. The percentage of CD4+ and CD8+ T cells expressing PD1 was dramatically reduced early during treatment with anti-PD1, in adequacy with the pharmacodynamics of anti-PD1,^23,24^ but this was observed on both R and NR patients (Figure 4(a)). This significant decrease was not due to masking of the PD1 epitope by the drug, as previously demonstrated,^25^ as PD1 was detected with a combination of an anti-PD1 antibody (clone 1.3.1) and an anti-human IgG4 that recognizes the drug (nivolumab). Of upmost importance, we observed a significant time-dependent reduction of the expression of HVEM on both CD4+ and CD8+ T cells exclusively in patients who responded to anti-PD1 (Figure 4(b)). In addition, the reduction of HVEM, but not PD1, expression in either CD4+ or CD8+ T cells, quantified as the difference of the percentage of cells expressing the marker at W12 vs baseline, was significantly different between R and NR patients (Figure 5). In contrast, the expression of its receptor BTLA^26^ was not modified by the treatment in either R or NR patients (Figure 4(c)). We also observed a decreased expression of CD69 on CD8+, but not CD4+ T cells in R patients after 3 months of treatment (W12) (Figures 4(d), 5), but also after only one infusion (W2) (Supplementary Figure S4a,b). In contrast, no difference between R and NR patients was noticed for the expression of CD137, TIGIT and PDL1 on CD4+ and CD8+ T cells (Supplementary Figure S3). Together, these results highlight evolution after three months of anti-PD1 treatment of HVEM and CD69 as markers that are significantly different between R and NR patients.

Whole blood CD4+ and CD8+ T cells from MM patients collected at different time points (inclusion, W2, W12) were subjected to FC “ICP/ICP-L panel” presented in Supplementary Table 1D) and segregated according to their response to anti-PD1 (R, NR). Modulation of PD1 (a), HVEM (b), BTLA (c) and CD69 (d) on total CD4+ and CD8+ T cells in the individual patients. 2-way ANOVA test was used to compare the evolution of each parameter during treatment in the two groups.

The modulation (difference between inclusion and W12) of each parameter (PD1, HVEM, CD69, CD137) was calculated and correlated to the response to anti-PD1. The median of each group was presented. R and NR patients were compared using Mann-Whitney U test.

### Enhancement of T cell polyfunctionality following treatment in responding patients

**Figure 6.**
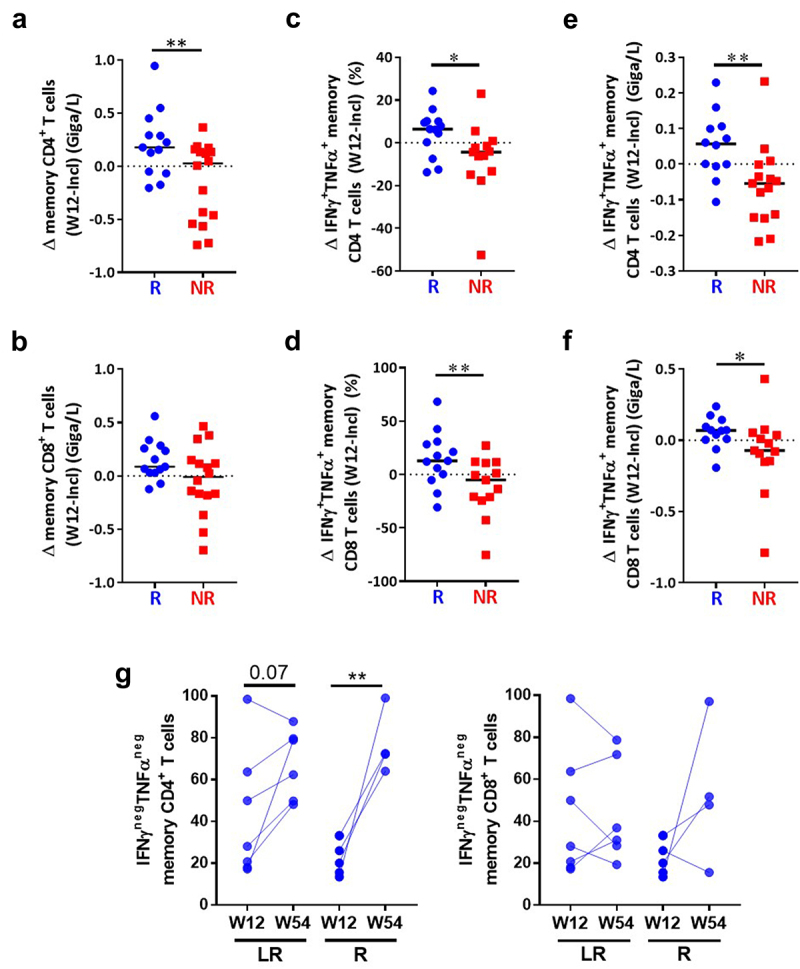
Increased memory T cell absolute number and polyfunctionality under treatment in responding patients.

We also examined the evolution of absolute numbers and polyfunctionality of memory CD4+ and CD8+ T cells during anti-PD1 treatment. While after a single injection of anti-PD1 (W2) memory T cell counts were not merely modified (Supplementary Figure S4c), we observed, after three months of treatment (W12), a significant increase in the absolute numbers of CD4+ (Figure 6(a)), but not CD8+ (Figure 6(b)) memory T cells in a majority of R patients and about half of the NR patients. In addition, the proportions of polyfunctional (IFN-γ^+^TNF-α^+^) CD4+ and CD8+ memory T cells increased at W12 as compared to inclusion in 76.9% (10/13) of R versus 23.1% (3/13) of NR patients for CD4+ T cells (Figure 6(c)) and 76.9% (10/13) versus 38.4% (5/13) for CD8+ T cells (Figure 6(d)). A similar tendency was already observed at W2 for CD4+ T cells (Supplementary Figure S4c) and CD8+ T cells (Supplementary Figure S4d). A significant treatment-induced increase of memory CD4+ and CD8+ T cell polyfunctionality was observed in R compared to NR patients at W12, both in terms of percentages (Figure 6(c,d)) and absolute numbers (Figure 6(e,f)), with a similar trend (*p* = .07) at W2 (Supplementary Figure S4d).

Within CD4 and CD8 memory T cells, the modulation (difference between inclusion and W12) of absolute number (a, b) and percentage (c, d) or number (e, f) of polyfunctional T cells was calculated and correlated to the response to anti-PD1. The median of each group was presented. R and NR patients were compared using Mann-Whitney U test. (g) Proportion of nonfunctional (IFN-γ^neg^TNF-α^neg^) memory CD4+ and CD8+ T cells responding MM patients (*n* = 10) segregated on R patients (R, *n* = 4) and long-term ones (LR, *n* = 6). Statistical analysis ANOVA-2.

Importantly, in R patients (*n* = 10) after one year of treatment, we intriguingly found that a significant proportion of memory CD4+ but not CD8+ T cells had a drastic reduced capacity to secrete cytokines in response to activation, as demonstrated by the strong increase in cells producing no cytokine that was particularly obvious in patients who were responders at W54 but relapsed after the treatment arrest (R)(*p* = .016) whereas only a trend (*p* = .07) was noticed for those who presented long-term response (LR) (Figure 6(g)).

Overall, these results suggest that a fraction of nonfunctional CD4+ and CD8+ T cells are reactivated during anti-PD1 treatment selectively in R patients, but long-term exposure to anti-PD1 may induce their terminal exhaustion instead that may favor relapse.

### Biomarkers combination allows to better discriminate responders from non-responders

One major challenge in immunotherapy is the lack of reliable parameters that can be used in clinical practice to predict anti-PD1 treatment efficacy prior to, or early during treatment, to adapt the therapeutic strategy. As *per se*, none of the individual parameters was able to firmly discriminate R from NR patients, we aimed to identify a composite biomarker defining response to anti-PD1 by integrating all individual blood immune parameters associated with clinical response, e.g. changes (W12-W0) in HVEM and CD69 and in absolute numbers and polyfunctional memory T cells. We included these parameters for both CD4+ and CD8+ T cells, whenever the parameter has been positively associated with one or the other population.

We first analyzed the possible correlation between these markers. The reduced HVEM expression during treatment on both CD8+ and CD4+ T cells were significantly correlated (*r* = 0.934, *p* < 10^−4^) and also respectively correlated with a decrease in CD69 expression in CD8+ T cells (*r* = 0.67, *p* = .0002) and a trend for decreased CD69 expression in CD4+ T cells (*r* = 0.38; *p* = .05) (Supplementary Figure S5). In contrast, the reduction of HVEM expression in either CD4+ or CD8+ T cells did not correlate with increase of their polyfunctionality nor their absolute number (Supplementary Figure S5). Moreover, the evolution of number of polyfunctional memory CD4+ and CD8+ T cells were highly correlated together (*r* = 0.795, *p* < 10^−4^) but were anti-correlated respectively with the evolution of CD69 on CD4+ (*r* = −0.985, *p* < 10^−4^) and on CD8+ (*r* = −0.998, *p* < 10^−4^) T cells (Supplementary Figure S5). Whereas tested independently, the selected parameters failed to accurately discriminate R from NR in ROC curves (Supplementary Figure S6a), the combination of all these parameters (Supplementary Figure S6b), gave good performance with an AUC of 0.82.

**Figure 7.**
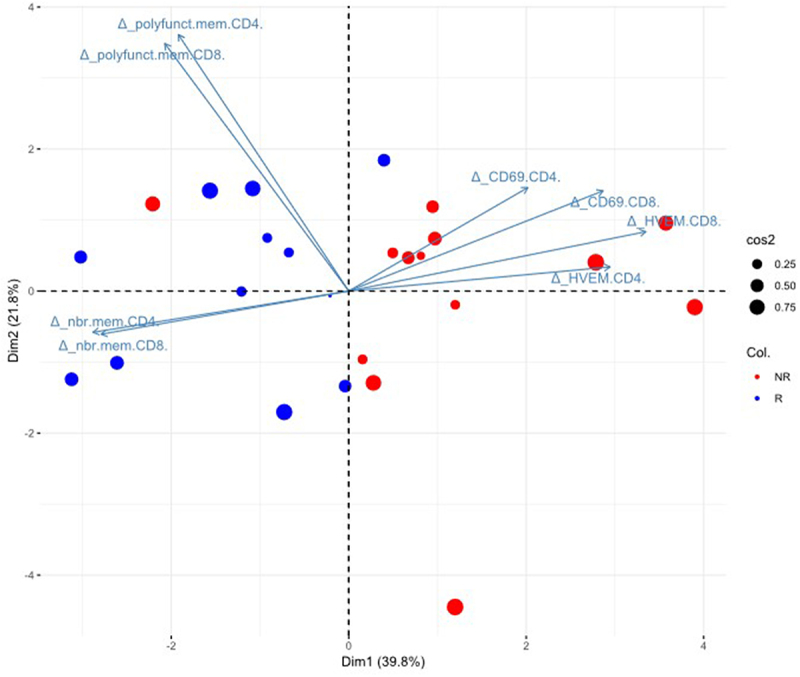
The biomarkers of interest selected in this study allow to discriminate responding from non-responding patients.

For patients for which all parameters (changes in percentage of HVEM and CD69 expression, absolute number and polyfunctionality) for either CD4+ or CD8+ T cells could be evaluated (24/29), a PCA combining all parameters was able to discriminate R from NR patients in 93.1% (24/26) of cases (Figure 7). All but one (patient #08) of the 13 R patients were correctly assigned and only 1/13 NR patients (patient #01) was misclassified.

PCA analysis including all the biomarkers significantly associated with response to treatment on CD4+ and/or CD8+ T cells. The weight of the variables from the PCA explaining the discrimination of R from NR are presented by the arrows.

## Discussion

In this study, we report an alteration of innate and adaptive blood immune cells in MM patients prior to initiation of second line anti-PD1 treatment, affecting the frequency and/or function of DC subsets, monocytes, NK cells and T cells. However, none of these baseline alterations was predictive of the response or resistance to anti-PD1. Whereas reduction of PD1 expression on T cells upon treatment did not correlate with clinical response, a clear decrease of HVEM expression on both CD4+ and CD8+ T cells was observed in responding patients, highlighting HVEM/BTLA as a potential resistance mechanism to anti-PD1 treatment. This was associated with a decreased proportion of CD69-expressing CD8+ T cells in R patients. Moreover, an increase of absolute number and polyfunctionality of memory CD4+ and CD8+ T cells also identified R patients. Furthermore, the combined evaluation of these different parameters together allowed to discriminate R from NR patients with a success rate of 93,1%.

## At baseline, MM patients present alterations of blood innate and adaptive immune cells compared to HD

DC, and in particular the cDC1 subset, absolute numbers appeared significantly reduced in MM patients compared to HD, in agreement with a recent report from Lee *et al*.^27^ In contrast, other innate immune cell populations such as NK cells or monocytes were not altered. cDC1 compartment, which excel in cross-presenting tumor antigens to CD8+ T cells,^28^ play a major role in the induction and maintenance of CD8+ T cell antitumor immunity^29^ and are critical for efficacy of various immunotherapies in murine models. Indeed, several studies have demonstrated that depletion of cDC1 strongly reduced the capacity of anti-PD1 treatment to control established tumors.^30–32^ Moreover, we recently reported the inefficacy of anti-PDL1 treatment in a phase I clinical trial when combined with pexidartinib (Daiichi-Sankyo), a tyrosine kinase inhibitor inhibiting CSF-1 R but also the differentiation of DC from bone marrow progenitors through its action on FLT3-L.^33^ In addition, while most DCs remained functional, except pDC that presented an increased capacity to produce TNFα, we observed a strong alteration of NK cell functionality and TNFα production by monocytes. Such alterations of NK cells and monocytes function have been previously reported by us and others in patients with metastatic tumors.^19,34^ IFN-γ secretion by blood NK cells measured in response to R848 requires crosstalk with monocyte and DC through IL-18 and IL-12,^35–37^ suggesting an alteration of this dialogue in patients that could limit their capacity to mount a potent antitumor immune response. Importantly, these alterations do not depend on the previous line of treatment (Supplementary Figure S7).

We also noticed a higher expression of ICPs (PD1, TIGIT, BTLA, CD69) on total CD4+ and CD8+ T cells in patients compared to HD, with the exception of CD73. This could partly result from the decrease of the naïve T cell compartment that was previously reported in advanced (stage III/IV) MM patients compared to HD.^27,38^ In addition, MM patients presented an increased polyfunctionality of memory CD8+ T cells that could reflect an ongoing systemic immune activation/immune response upon tumor progression.

Prior to anti-PD1 treatment onset, we observed the expression of HVEM and its receptor BTLA on a larger proportion of CD4+ and CD8+ T cells in MM patients compared to HD. HVEM is expressed on the surface of immune cell subsets, including T cells (for review),^39^ on which it acts either as an ICP-L through BTLA^26^ and CD160^40^ receptors to deliver an inhibitory signal or as a receptor of LIGHT and Lymphotoxin-α to transduce a positive signal.^41^ Demerlé *et al* recently reported higher HVEM expression on less differentiated T cell subsets (naïve (T_N_) and central memory (T_CM_)) compared to effector memory (T_EM_), and early activated T cells.^42^ The reduced proportion of T_N_ and the absence of modulation of T_CM_ subsets observed in MM patients compared to HD strongly argue in favor of an activated status of CD4+ and CD8+ T cells as illustrated by the high expression of PD1 and TIGIT.

## Decreased proportions of blood HVEM- and CD69-expressing T cells during treatment discriminate responding from non-responding patients

PERLS^21^ and NLR^22^ scores or high blood frequency of PD1+ CD8+ T cells^15^ previously proposed as biomarkers associated with response to anti-PD1 in solid tumors at time of treatment initiation, failed to correlate with response in the present study. Moreover, T cell absolute numbers and individual inhibitory ICP expression on T cells did not correlate with response. In contrast, a reduced proportion of polyfunctional memory CD4+ T cells characterized patients who respond to anti-PD1.

Upon treatment, we observed a strong reduction of PD1 expression on both CD4+ and CD8+ T cells, in accordance with the literature,^24^ but this effect remained independent of the clinical response. Of great interest, we highlighted a decrease of HVEM expression on CD4+ and CD8+ T cells under treatment (at W12 after treatment onset) only in R patients, whereas expression of its receptor BTLA was not modified. This was associated with a decreased proportion of CD69-expressing CD8+ T cells in R patients. This decrease could reflect a modulation in the state of differentiation of CD4+ and CD8+ T cells, as HVEM expression is downregulated during T cell differentiation.^42^ However, no association between HVEM levels in T cells and the frequency of memory T cell subsets was observed (Supplementary Figure S8a). Alternatively, as a ligand for BTLA,^43^ that belongs to the CD28 family and shares structural similarities with PD1 and CTLA-4^44^ with an ITIM motif in its cytoplasmic tail, HVEM may be part of a resistance mechanism inhibiting both proliferation and cytokines production by T cells through cis-inhibition.^45^ While the number of CD4+ and CD8+ memory T cells increased after 3 months of anti-PD1 treatment in R patients, no significant modulation in the different subsets (T_CM_, T_EM_, T_EMRA_) absolute numbers was noticed when comparing R to NR patients (Supplementary Figure 8b) thus excluding a link between HVEM downregulation and T cell differentiation state in this trial. In addition, BTLA expression was not significantly modulated upon treatment in both R and NR patients, suggesting that the cis-inhibition through HVEM may play an important role in the inhibition of T cells proliferation and function (cytokine co-production) in NR patients. Therefore, the high expression of BTLA and HVEM on CD4+ and CD8+ T cells might act as an additional inhibitory pathway acquired in the tumor microenvironment to impair anti-tumor immune response. One limit of this study is the lack of analysis of the other HVEM receptors, such as CD160 that can initiate an inhibitory signal in T cells^40^ or LIGHT and Lymphotoxin-α involved in T cell activation upon ligation with HVEM.^41^ Indeed, the reduction of HVEM expression could also result from interaction with LIGHT in cis or in trans as it is expressed on activated T cells but also on monocytes, and activated B cells (for review^46^).

This reduced HVEM expression on both CD4+ and CD8+ T cells in anti-PD1 responsive patients together with the blockade of PD1/PDL1 axis probably participate to their reactivation. In this context, recent *in vitro* data revealed the ability of a neutralizing anti-HVEM antibody (clone HVEM18–10) to enhance both activation (CD25 upregulation) and proliferation of CD4+ and CD8+ T cell by blocking the function of HVEM in cis.^42^ Moreover, anti-HVEM antibody synergizes *in vitro* with anti-PDL1 blockade to enhance T cell responses against lung cancer cell line expressing both PDL1 and HVEM.^42^ Yet, as neither CD25 nor Ki67 were integrated in our FC panels, we were not able to confirm that HVEM downregulation was correlated with reactivation of T cells.

We observed association between a decrease in CD69 expression in CD8+ T cells and response to anti-PD1 treatment. Interestingly, we noticed that this decrease strongly correlated with that of HVEM expression observed in R patients. Although CD69 is considered as an activation marker, this could suggest that anti-PD1 treatment selectively reinvigorate T cells with low HVEM levels leading to their recruitment in the tumor in R patients. As CD69 is also a tissue residency marker,^47^ the recruitment in the tumor of activated T cell could explain the decrease of CD69+ circulating T cells observed in R patients.

## Increased memory T cell polyfunctionality during treatment discriminate responding from non-responding patients

Finally, we demonstrated, for the first time, that an increased number of polyfunctional blood CD4+ and CD8+ memory T cells at W12 after the onset of anti-PD1 treatment (with a similar trend at W2), was associated with the response to anti-PD1 treatment and evolved in parallel to the HVEM decrease. This increase resulted from both the increase of absolute number and polyfunctionality of these memory CD4+ and CD8+ T cells. This result is in adequacy with the mode of action of anti-PD1 known to amplify memory T cells and reactivate their functions (for review^20^). A recent report from Lao et al.^48^ reported, in metastatic NSLCC patients, an increased cytokine secretion (IL-2, IFNγ, TNFα) in responders two weeks after the initiation of anti PD1 treatment but didn’t investigate the polyfunctionality of these T cells.

In contrast, Kim et al.^49^ recently reported, in NSCLC and thymic epithelial tumors, an absence of modulation of effector CD4+ and CD8+ T cell cytokine production (IFN-γ or TNF-α) upon anti-CD3 stimulation early after anti-PD1 treatment initiation (day 7) but they did neither evaluate coproduction of cytokines nor their long-term (W12) modulation. However, they identified an association between increased TNF-α secretion by blood CD4 and CD8 T cells and the development of immune-related adverse events that could rely on anti-PD1 biological efficacy.

However, it has to be noticed that, in responders, the continuous treatment with anti-PD1 during 1 year leads to an increase of nonfunctional memory CD4+ and CD8+ T cells that could result from their chronic activation leading to functional exhaustion, as previously described in chronic viral infections.^50^ Interestingly this dysfunction is more pronounced in patients who finally relapsed than in long-term responders, suggesting that the loss of function in blood memory T cells could participate to relapse. It could be important to evaluate intermittent anti-PD1 strategy to prevent this functional alteration. However, a recent real-life study in MM reported the maintenance of long-lasting responses after anti-PD1 interruption and highlighted that 70.6% of recurrences occurred in patients who were not experiencing complete response at time of treatment discontinuation.^51^

Whereas taken independently each significant change between inclusion and W12 (HVEM, CD69, number of memory T cells, T cell polyfunctionality) is insufficient to segregate R from NR patients with confidence, their combination well discriminated R from NR in our cohort, with only two miss-classified patients. We did not find any specific characteristic of blood immune cells nor patient clinical features that could explain miss-classification of these two patients.

Our results also suggest that targeting HVEM/BTLA pathway could be of interest to improve anti-PD1 efficacy and such drugs are currently under development. In particular, two phase I trials evaluated, in patients with advanced cancer refractory to standard therapies, the impact of anti-BTLA mAbs (NCT04137900, NCT04773951)) that block BTLA/HVEM interaction. Results from the dose-expansion part of the NCT04137900 study (19 patients) highlighted a durable partial response (>12 months) for a MM patient who previously progressed on anti-PD1 and BRAF/MEK inhibitors treatment.^52^

## Supplementary Material

Dalle et al supplementary Figures clean.pdf

## Data Availability

The datasets used during the current study are available from the corresponding author on reasonable request.
