# Supplementary material for: Modulation of blood T cell polyfunctionality and HVEM/BTLA expression are critical determinants of clinical outcome in anti-PD1-treated metastatic melanoma patients
Source: Oncoimmunology. 2024 Jun 26;13(1):2372118. doi: 10.1080/2162402X.2024.2372118 (PMC11210932; doi:10.1080/2162402X.2024.2372118)

**Modulation of Blood-T cell polyfunctionality and HVEM/BTLA expression are critical determinants of clinical outcome in anti-PD1-treated metastatic melanoma patients**

Stéphane Dalle [1,3]*, Estelle Verronese[2]*, Axelle N'Kodia[2]*, Christine Bardin[2], Céline Rodriguez [2,3], Thibault Andrieu[2,3], Anais Eberhardt[1,3], Gabriel Chemin[3], Uzma Hasan[2], Myrtille Le-Bouar[1], Julie Caramel[3], Mona Amini-Adle[1], Nathalie Bendriss-Vermare[2,3], Bertrand Dubois[2,3], christophe Caux[2,3¥], Christine Ménétrier-Caux[2,3¥] (*co-first authorship, ¥Co-last authorship)

# Supplementary Material

**Supplementary Table 1: Flow cytometry panels used for phenotypic analyses**
Flow cytometry panels with clones and producers ([1] BD Biosciences, [2] Myltenyi Biotec, [3] Biolegend, [4] Biotechne, [5] Southern Biotechnologies, [6] e-Bioscience) to identify monocyte subsets (**A**), DC subsets (**B**), $CD4^+$and $CD8^+$ T cell subsets ($T_N$, $T_{CM}$, $T_{EM}$, $T_{EMRA}$) (**C**), and expression of ICP and ICP-L on $CD4^+$ and $CD8^+$ T cells (**D**).

**Supplementary Table 2: Patients characteristics of the PAIR cohort and associated prognostic values.**

**Supplementary Table 3: None of biological parameters analyzed except memory CD4+ T cells polyfunctionality was correlated to response to anti-PD1.** All investigated parameters (absolute numbers (**A**), ICP/ICP-L expression on total CD4+ and CD8+ T cells (**B**) and functionality of innate (monocytes, NK cells, DC subsets) and memory CD4+ and CD8+ T cells (**C**) at time of inclusion were correlated with response to anti-PD1.

**Supplementary Figure 1: Flow cytometry panels to investigate, on whole blood, the modulation of immune cell subsets phenotype and activation status during treatment with anti-PD1. A-** Panels and gating strategy example for the analysis of immune cell subsets (B cells, monocytes, DC and T cell subsets) absolute numbers (**A**) and ICP/ICP-L expression on total CD4+ and CD8+ T cells (**B**) on EDTA-collected whole blood samples.

**Supplementary Figure 2: Flow cytometry panels to investigate, on whole blood, the modulation of immune cell subsets functionality during treatment with anti-PD1.** Panels and gating strategy example for the analysis of innate (monocytes, NK cells and DC subsets) (**A**) and adaptive (**B**) blood immune cell on heparin-collected whole blood samples after short-term *in vitro* reactivation respectively with TLR7/8 ligand and PMA/ionomycin (T cells) in the presence of a secretion inhibitor.

**Supplementary Figure 3: PDL1, CD137 and TIGIT expression in melanoma patients are not modulated by the response to anti-PD1.** Whole blood CD4+ and CD8+ T cells from melanoma patients collected at different time points (inclusion, W2, W12) were subjected to flow cytometry "ICP/ICP-L panel" presented in **supplementary Fig. 1A**) and segregated according to their response to anti-PD1 (R, NR). Modulation of PDL-1 (**A**), CD137 (**B**), and TIGIT (**D**) on total CD4+ and CD8+ T cells.

**Supplementary Figure 4: Modulation of HVEM, CD69 expression as well as absolute number and polyfunctionality of CD4+ and CD8+ memory T cells after one cycle of anti-PD1 according to response.** Within CD4 and CD8 memory T cells, the modulation (difference (D) between inclusion and W2) of the frequency of $CD69^+$ (**A**) and $HVEM^+$ (**B**), the absolute number (**C**) and percentage (**D**) of polyfunctional T cells was calculated and correlated to the response to anti-PD1. The median of each group was presented. patients and HD were compared using Mann-Whitney U test.

**Supplementary Figure 5: Matrix of Pearson's correlation coefficients between all parameters of evolution (W12-incl) characterized as associated with response to anti-PD1 in MM patients.** Points are colored according to the value of the Pearson's correlation coefficient and sized according to the inverse of the p-value.

**Supplementary Figure 6:** ROC curves for each parameter of interest tested individually **(A)** or in combination **(B)**.

**Supplementary Figure 7: Altered functionality of innate immune cells did not relate of the previous line of treatment.** MM patients were segregated according to their first line of treatment (BRAF-i (C1) and anti-CTLA-4 (C2)) and the proportion of TNF$\alpha$-expressing pDC and monocytes as well as IFN-$\gamma$-expressing NK cells after short-term activation with R848 was evaluated. Patients from C1 (Vemurafenib) and C2 (Ipilimumab) and HD were compared by 1-way ANOVA.

**Supplementary Figure 8: Evolution of CD4 and CD8 T cell subsets and response to anti-PD1.** Proportion of HVEM+ CD4+and CD8+ T cells at inclusion and W12 did not correlate with the absolute number of memory cells and T cell subsets ($T_N$, $T_{CM}$, $T_{EM}$, $T_{EMRA}$) (**A**). No significant difference in evolution (W12-incl) of CD4+ or CD8+ T cell subsets according to response to anti-PD1 (**B**).

# Supplementary Table 1

**A**

| | Monocyte subsets | |
|---|---|---|
| **Fluorochrome** | **Marker** | **Clone** |
| **BV421** | [1]CD3/[1]CD7/[1]CD15 | UCHT1/MT701/MC480 |
| **BV510** | [1]CD16 | 3G8 |
| **PerCP** | [2]CD14 | TÜK4 |
| **PE-Vio770** | [2]CD19 | LT19 |
| **APC-Cy7** | [3]Zombie NIR | NA |

**B**

| | DC subsets | |
|---|---|---|
| **Fluorochrome** | **Marker** | **Clone** |
| **BV421** | [7]Lineage-1 | see[7] |
| **BV510** | [3]HLA-DR | L243 |
| **PerCP** | [3]CD1c (BDCA1) | L161 |
| **PE-Cy7** | [3]CD11c | Bu15 |
| **APC** | [2]CD303 / [2]CD141 | AC144/AD5-14H12 |
| **APC-Cy7** | [3]Zombie NIR | NA |

**C**

| | T cell subsets | | | |
|---|---|---|---|---|
| **Fluorochrome** | **Marker** | **Clone** | **Marker** | **Clone** |
| **BB515** | [1]CD3 | UCHT1 | [1]CD3 | UCHT1 |
| **BV421** | [1]CD27 | M-T271 | [1]CD278 (ICOS) | DX29 |
| **BV510** | [1]CD28 | CD28.2 | [1]CD185 (CXCR5) | RF8B2 |
| **PE** | [4]CCR7 | 150503 | [2]CD279/[5]IgG4 | PD1.3.1.3/HP6025 |
| **PerCP** | [2]CD4 | M-T466 | [2]CD4 | M-T466 |
| **PE-VIO770** | [2]CD8 | BW135/80 | [2]CD8 | BW135/80 |
| **APC** | [1]CD45RA | HI100 | [2]FOXP3 | 3G3 |
| **APC-Cy7** | [3]Zombie NIR | NA | [3]Zombie NIR | NA |

**D**

| | T cells Isotype | | T cells ICP1 | | T cells ICP2 | | T cells ICP3 | | T cells ICP4 | | T cells ICP5 | |
|---|---|---|---|---|---|---|---|---|---|---|---|---|
| **Fluorochrome** | **Marker** | **Clone** | **Marker** | **Clone** | **Marker** | **Clone** | **Marker** | **Clone** | **Marker** | **Clone** | **Marker** | **Clone** |
| **BB515** | [1]CD3 | UCHT1 | [1]CD3 | UCHT1 | [1]CD3 | UCHT1 | [1]CD3 | UCHT1 | [1]CD3 | UCHT1 | [1]CD3 | UCHT1 |
| **BV510** | [1]CD56 | NCAM16.2 | [1]CD56 | NCAM16.2 | [1]CD56 | NCAM16.2 | [1]CD56 | NCAM16.2 | [1]CD56 | NCAM16.2 | [1]CD56 | NCAM16.2 |
| **PerCP** | [2]CD4 | M-T466 | [2]CD4 | M-T466 | [2]CD4 | M-T466 | [2]CD4 | M-T466 | [2]CD4 | M-T466 | [2]CD4 | M-T466 |
| **PE-Vio770** | [2]CD8 | BW135/80 | [2]CD8 | BW135/80 | [2]CD8 | BW135/80 | [2]CD8 | BW135/80 | [2]CD8 | BW135/80 | [2]CD8 | BW135/80 |
| **PE** | [1]Mouse IgG1 | MOPC-21 | [2]CD279 + [5]IgG4 | PD1.3.1.3 / HP6025 | [6]TIGIT | MBSA43 | [1]BTLA | J168-540 | | | | |
| **APC** | [1]Mouse IgG1 | MOPC-21 | [1]PDL-1 | MIH1 | [6]CD73 | AD2 | [2]HVEM | REA247 | [1]CD137 | 4B4-1 | [2]CD69 | FN50 |
| **APC-Cy7** | [3]Zombie NIR | NA | [3]Zombie NIR | NA | [3]Zombie NIR | NA | [3]Zombie NIR | NA | [3]Zombie NIR | NA | [3]Zombie NIR | NA |

*Supplementary Figure 1*

# 1A

## Monocyte subsets Panel

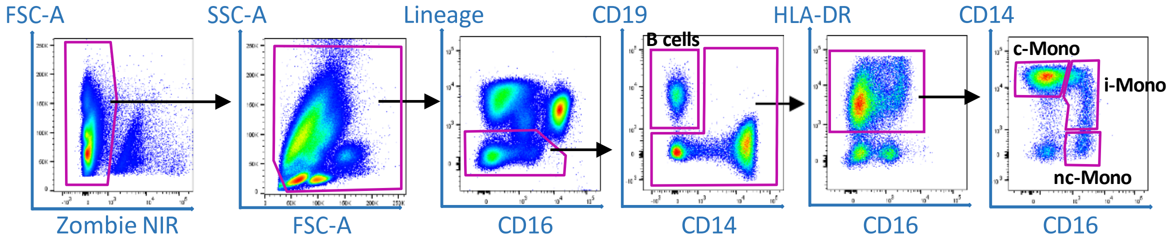


## DC subsets Panel

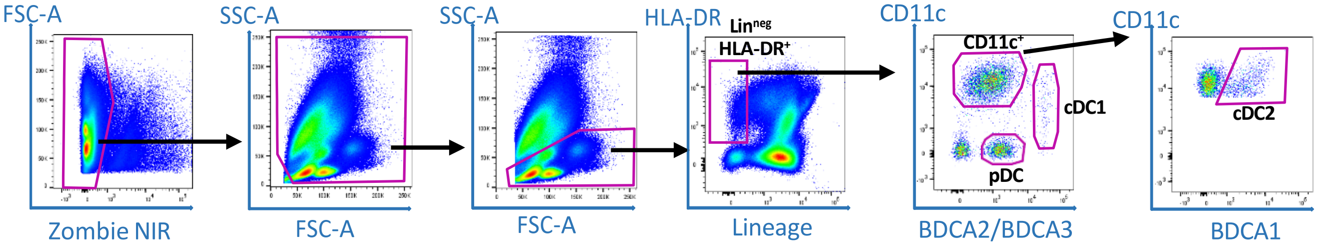


## T cell subsets Panel

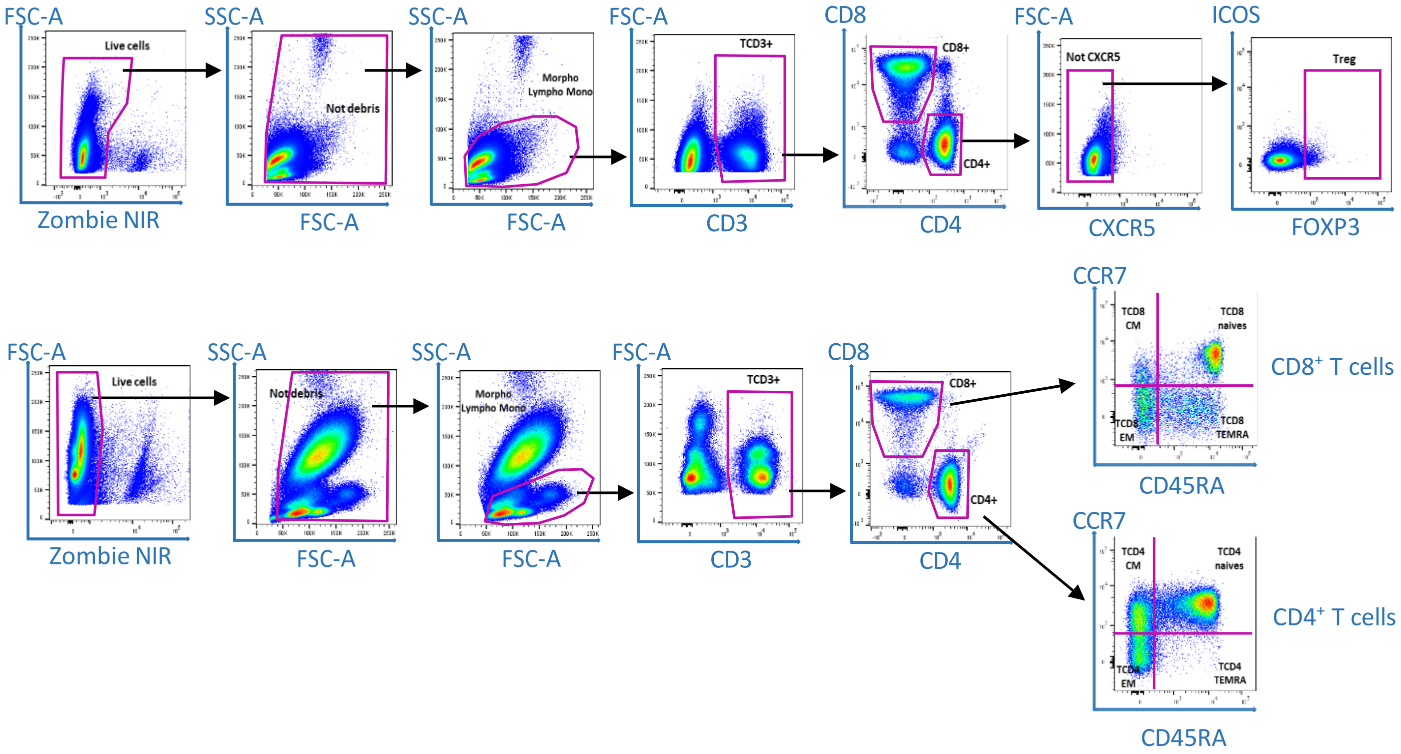

# Supplementary Figure 1

## 1B

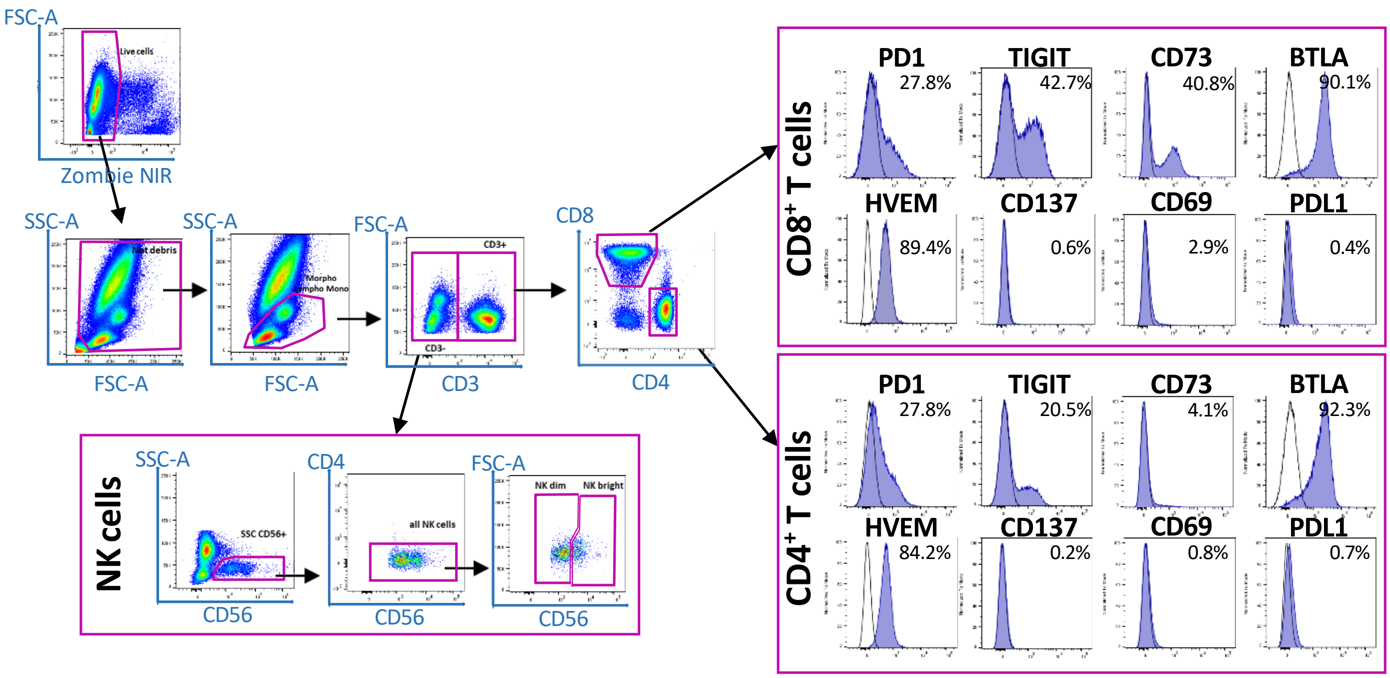

*Supplementary Figure 2*

**2A**

| | Panel Innate Cell Function | | | |
|---|---|---|---|---|
| | **Marker** | **Fluorochrome** | **Clone** | **Manufacturer** |
| **Membrane** | Zombie Yellow | BV570 | NA | BioLegend |
| | CD3 | V500 | UCHT1 | BD Biosciences |
| | CD14 | V500 | M5E2 | BD Biosciences |
| | CD15 | V500 | HI98 | BD Biosciences |
| | CD19 | V500 | HIB19 | BD Biosciences |
| | CD11c | PerCP-Cy5.5 | Bu15 | BioLegend |
| | CD16 | ECD | 3G8 | Beckman Coulter |
| | CD56 | BV711 | NCAM16.2 | BD Biosciences |
| | HLA-DR | APC-H7 | L243 | BD Biosciences |
| | BDCA1 | PE-Cy7 | L161 | BioLegend |
| | BDCA2 | APC | AC144 | Miltenyi Biotec |
| | BDCA3 | APC | AD5-14H12/REA674 | Miltenyi Biotec |
| **Intra-cytoplasmic** | IL-12p40 | PE | C11.5 | BD Biosciences |
| | INFα | FITC | 4S.B3 | Miltenyi Biotec |
| | IFNγ | BV421 | 4S.B3 | BioLegend |
| | TNFα | Alexa Fluor 700 | MAb11 | BD Biosciences |

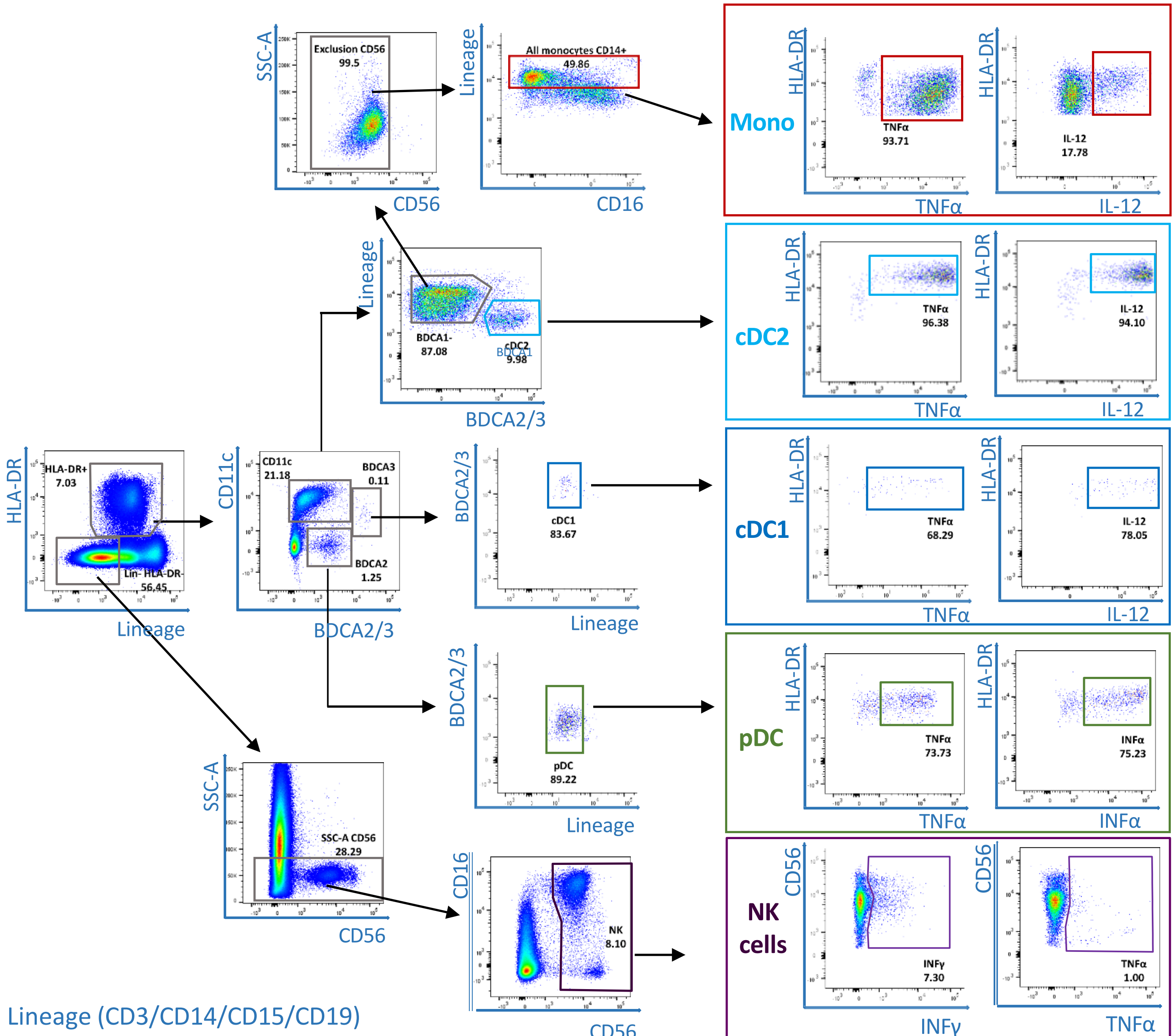

# 2B

| | Panel Innate Cell Function | | | |
|---|---|---|---|---|
| | **Marker** | **Fluorochrome** | **Clone** | **Manufacturer** |
| **Membrane** | Zombie Yellow | BV570 | NA | BioLegend |
| | CD3 | V500 | UCHT1 | BD Biosciences |
| | CD14 | V500 | M5E2 | BD Biosciences |
| | CD15 | V500 | HI98 | BD Biosciences |
| | CD19 | V500 | HIB19 | BD Biosciences |
| | CD11c | PerCP-Cy5.5 | Bu15 | BioLegend |
| | CD16 | ECD | 3G8 | Beckman Coulter |
| | CD56 | BV711 | NCAM16.2 | BD Biosciences |
| | HLA-DR | APC-H7 | L243 | BD Biosciences |
| | BDCA1 | PE-Cy7 | L161 | BioLegend |
| | BDCA2 | APC | AC144 | Miltenyi Biotec |
| | BDCA3 | APC | AD5-14H12/REA674 | Miltenyi Biotec |
| **Intra-cytoplasmic** | IL-12 p40 | PE | C11.5 | BD Biosciences |
| | INFα | FITC | 4S.B3 | Miltenyi Biotec |
| | IFNγ | BV421 | 4S.B3 | BioLegend |
| | TNFα | Alexa Fluor 700 | MAb11 | BD Biosciences |

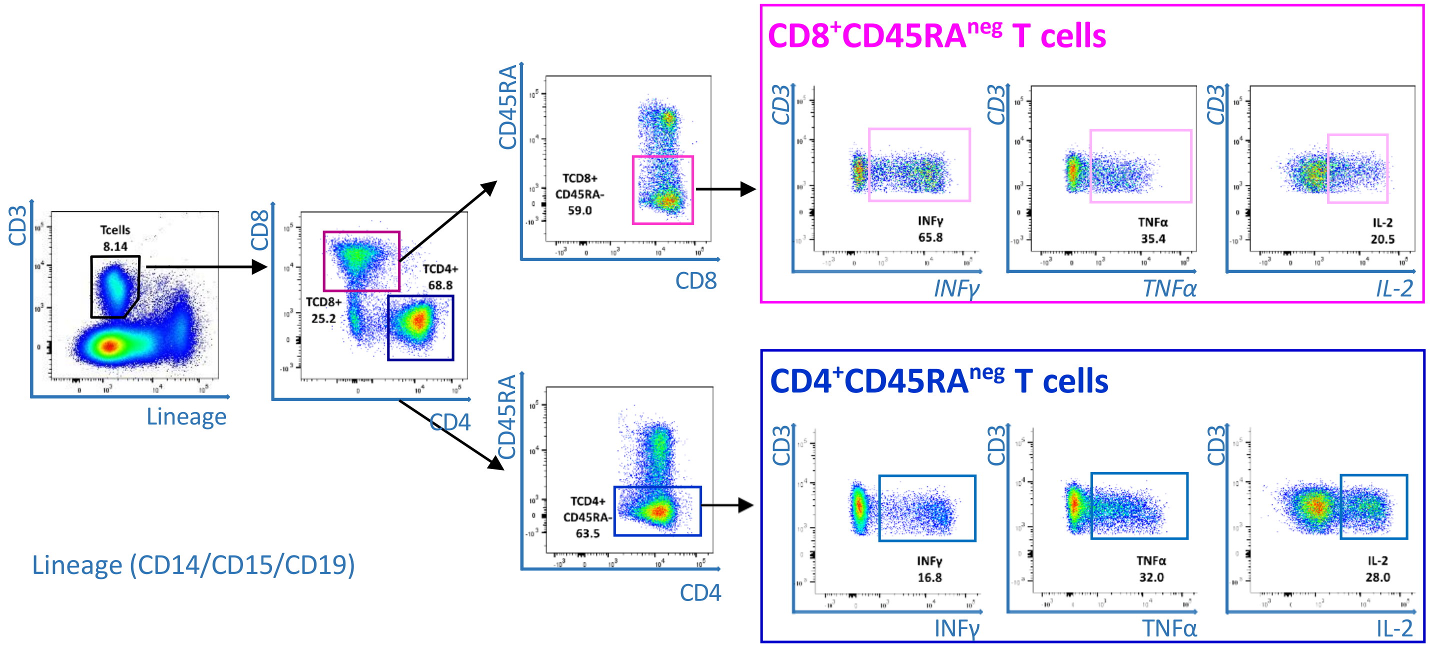

# *Supplementary Table 2*

| Cohort | % (min-max) | 1-year clinical response | | Statistical test<br>P value |
|---|---|---|---|---|
| | | R<br>13 (45%) | NR<br>16 (55%) | |
| **Age** | | | | |
| Years, median (range) | 61 (33-92) | 65 (33-82) | 57 (39-92) | T-test<br>ns, p=0,4824 |
| **Sex** | | | | |
| Female, n (%) | 10 (35) | 5 (38) | 5 (31) | Fisher's exact test<br>ns, p=0,7141 |
| Male, n (%) | 19 (65) | 8 (62) | 11 (69) | |
| **BRAF/NRAS mutational status** | | | | |
| BRAF, n (%) | 18 (62) | 6 (46) | 12 (75) | $X^2$ test<br>ns, p=0,1465 |
| NRAS, n (%) | 2 (7) | 2 (15) | 0 (0) | |
| Non-mutated, n (%) | 9 (31) | 5 (39) | 4 (25) | |
| **ECOG performance status** | | | | |
| 0, n (%) | 15 (52) | 8 (61) | 7 (44) | $X^2$ test<br>ns, p=0,4258 |
| 1, n (%) | 9 (31) | 3 (23) | 6 (37) | |
| 2, n (%) | 4 (14) | 1 (8) | 3 (19) | |
| ND, n (%) | 1 (3) | 1 (8) | 0 (0) | |
| **Breslow (at diagnosis)** | | | | |
| Presence, n (%) | 21 (72) | 11 (85) | 10 (62) | T-test<br>ns, p=0,9816 |
| Absence, n (%) | 8 (28) | 2 (15) | 6 (38) | |
| mm, median (range) | 3.6 (0.7-25.4) | 3.6 (0.7-18) | 3,65 (1.5-25.4) | |
| **Ulceration** | | | | |
| Yes, n (%) | 13 (45) | 7 (54) | 6 (38) | **$X^2$** test<br>**p=0,0477** |
| No, n (%) | 5 (17) | 4 (31) | 1 (6) | |
| ND, n (%) | 11 (38) | 2 (15) | 9 (56) | |
| **Brain metastases** | | | | |
| Yes, n (%) | 7 (24) | 2 (15) | 5 (31) | Fisher's exact test<br>ns, p=0,4100 |
| No, n (%) | 22 (76) | 11 (85) | 11 (69) | |
| **LDH** | | | | |
| <ULN, n (%) | 12 (41) | 6 (46) | 6 (38) | $X^2$ test<br>ns, p=0,1572 |
| >ULN, n (%) | 7 (24) | 1 (8) | 6 (38) | |
| ND, n (%) | 10 (35) | 6 (46) | 4 (25) | |
| **1$^{st}$ line treatment** | | | | |
| anti-BRAF +/- anti-MEK, n (%) | 18 (62) | 6 (46) | 12 (75) | Fisher's exact test<br>ns, p=0,1426 |
| Ipilimumab, n (%) | 11 (38) | 7 (54) | 4 (25) | |

NA = not applicable
ND = not determined
ULN = upper limit of normal = 246 U/L
ns : not signifcant

# *Supplementary Table 3*

**A**

| | | populations | Responders median (min-max) | Non Responders median (min-max) | p value |
|---|---|---|---|---|---|
| **Absolute numbers (Giga/L)** | **Mono** | c-monocytes | 0,307 (0,146-1,219) | 0,3081 (0,105-0,918) | 0,980 |
| | | nc-monocytes | 0,014 (0,005-0,126) | 0,0112 (0,003-0,046) | 0,347 |
| | | infl-monocytes | 0,229 (0,095-0,505) | 0,1561 (0,034-0,379) | 0,236 |
| | **DC** | cDC1 ($x10^{-1}$) | 0,003 (0,002-0,016) | 0,004 (0,001-0,014) | 0,774 |
| | | cDC2 ($x10^{-1}$) | 0,042 (0,020-0,230) | 0,041 (0,015-0,143) | 0,499 |
| | | pDC ($x10^{-1}$) | 0,053 (0,027-0,210) | 0,035 (0,002-0,183) | 0,097 |
| | | NK cells | 0,164 (0,030-0,615) | 0,176 (0,044-0,945) | 0,731 |
| | | B cells | 0,158 (0,064-0,351) | 0,117 (0,040-0,763) | 0,112 |
| | **T cells** | $CD3^+$ T cells | 1,561 (0,407-2,065) | 1,469 (0,206-2,225) | 0,631 |
| | | $CD4^+$ T cells | 1,06 (0,244-1,547) | 0,809 (0,087-1,543) | 0,428 |
| | | $CD4^+ T_N$ | 0,148 (0,001-0,559) | 0,164 (0,048-0,535) | 0,904 |
| | | $CD4^+ T_{CM}$ | 0,169 (0,002-0,385) | 0,171 (0,033-0,324) | 0,341 |
| | | $CD4^+ T_{EM}$ | 0,223 (0,119-0,759) | 0,267 (0,047-0,722) | 0,792 |
| | | $CD4^+ T_{EMRA}$ | 0,05 (0,018-0,106) | 0,084 (0,007-0,359) | 0,683 |
| | | Treg | 0,021 (0,004-0,044) | 0,012 (0,001-0,095) | 0,296 |
| | | total | 0,374 (0,124-0,706) | 0,466 (0,098-1,331) | 0,222 |
| | | $CD8^+ T_N$ | 0,026 (0,002-0,208) | 0,044 (0,003-0,123) | 0,589 |
| | | $CD8^+ T_{CM}$ | 0,012 (0,004-0,029) | 0,014 (0,004-0,404) | 0,567 |
| | | $CD8^+ T_{EM}$ | 0,07 (0,008-0,300) | 0,113 (0,039-0,643) | 0,222 |
| | | $CD8^+ T_{EMRA}$ | 0,12 (0,039-0,335) | 0,154 (0,035-0,542) | 0,299 |

**B**

| | | populations | Responders median (min-max) | Non Responders median (min-max) | p value |
|---|---|---|---|---|---|
| **ICP ICP/L expression on T cells** | **$CD4^+$** | $PD1^+$ T cells | 31,69 (17,23-48,16) | 32,13 (9,36-58,73) | 0,981 |
| | | $TIGIT^+$ T cells | 20,17 (14,67-30,48) | 19,53 (10,49-32,36) | 0,567 |
| | | $CD73^+$ T cells | 8,32 (0,38-21,09) | 7,21 (1,32-20,66) | 0,697 |
| | | $BTLA^+$ T cells | 86,6 (57,27-92,57) | 85,67 (76,9-95,31) | 0,567 |
| | | $HVEM^+$ T cells | 86,84 (49,98-99,21) | 76,73 (41,55-95,27) | 0,217 |
| | | $PDL1^+$ T cells | 0,88 (0,44-1,63) | 1,01 (0,14-10,79) | 0,631 |
| | **$CD8^+$** | $PD1^+$ T cells | 6,91 (7,46-63,3) | 30,3 (12,79-61,2) | 0,396 |
| | | $TIGIT^+$ T cells | 44,56 (16-76,57) | 40,09 (13,16-58,56) | 0,478 |
| | | $CD73^+$ T cells | 30,62 (7,43-53,95) | 22,97 (12,25-48,21) | 0,721 |
| | | $BTLA^+$ T cells | 64,21 (32,09-90,12) | 62,16 (38,02-92,77) | 0,873 |
| | | $HVEM^+$ T cells | 88,77 (69,64-99,63) | 72,8 (40,53-96,25) | 0,102 |
| | | $PDL1^+$ T cells | 0,64 (0,26-1,68) | 0,59 (0,22-2,69) | 0,697 |
| **Score** | | PERLS score | 1,13 (0,31-1,95) | 0,87 (0,49-3,79) | 0,301 |
| | | NLR | 2,71 (248-274) | 3,10 (2,63-3,32) | 0,288 |

**C**

| | | populations | Responders median (min-max) | Non Responders median (min-max) | p value |
|---|---|---|---|---|---|
| **Blood immune cell functionality** | **Mono** | IL-12p40+ Monocytes | 19,99 (8,93-33,29) | 20,33 (7,50-37,76) | 0.829 |
| | | TNFα+ Monocytes | 65,65 (39,43-80,47) | 66,14 (42,98-93,11) | 0.673 |
| | **NK** | IFNψ+ NK cells | 3,95 (0,80-19,01) | 3,20 (0-15,47) | 0.555 |
| | | TNFα+ NK cells | 1,65 (0,15-10,68) | 1,31 (0,17-23,09) | 0.650 |
| | **DC** | IFN/∴+ cDC1 | 21,31 (0,05-63,85) | 34,49 (14,85-64,8) | 0.109 |
| | | TNFα+ cDC1 | 60,18 (0-83,54) | 70,26 (11,7-95,46) | 0.673 |
| | | IL12p40+ cDC2 | 84,46 (61,54-95,62) | 91,93 (59,71-100) | 0.138 |
| | | TNFα+ cDC2 | 95,49 (51,28-98,26) | 94,91 (72,17-99,33) | 0.650 |
| | | IFNα+ pDC | 59,07 (27,24-69,18) | 62,84 (33,68-69,28) | 0.370 |
| | | TNFα+ pDC | 86,04 (70,1-96,16) | 92,01 (65,08-98,28) | 0.130 |
| | **T cells** | $IFN\psi^+$ memory CD4 T cells | 14,23 (0,13-62,53) | 22,32 (6,64-63,47) | ***0.041*** |
| | | $TNF\alpha^+$ memory CD4 T cells | 36,14 (1,16-84,16) | 41,65 (14,44-88,35) | 0.155 |
| | | $IL\text{-}2^+$ memory CD4 T cells | 21,31 (0,05-63,85) | 34,49 (14,95-64,8) | 0.109 |
| | | $IFN\psi^+$ memory CD8 T cells | 50,32 (3,00-87,86) | 62,58 (17,94-94,97) | *0.064* |
| | | $TNF\alpha^+$ memory CD8 T cells | 36,14 (1,13-84,16) | 41,65 (14,44-88,35) | 0.155 |
| | | $IL\text{-}2^+$ memory CD8 T cells | 13,56 (0,13-38,12) | 13,84 (6,86-28,62) | 0.611 |
| | | $IFN\psi^+TNF\alpha^+$ memory CD4 T cells | 9,11 (0,019-60,72) | 19,63 (4,19-59,96) | ***0.036*** |
| | | $IFN\psi^+TNF\alpha^+$ memory CD8 T cells | 5,33 (3,7-12,8) | 6,65 (3-14,5) | 0.130 |

*Supplementary Figure 3*

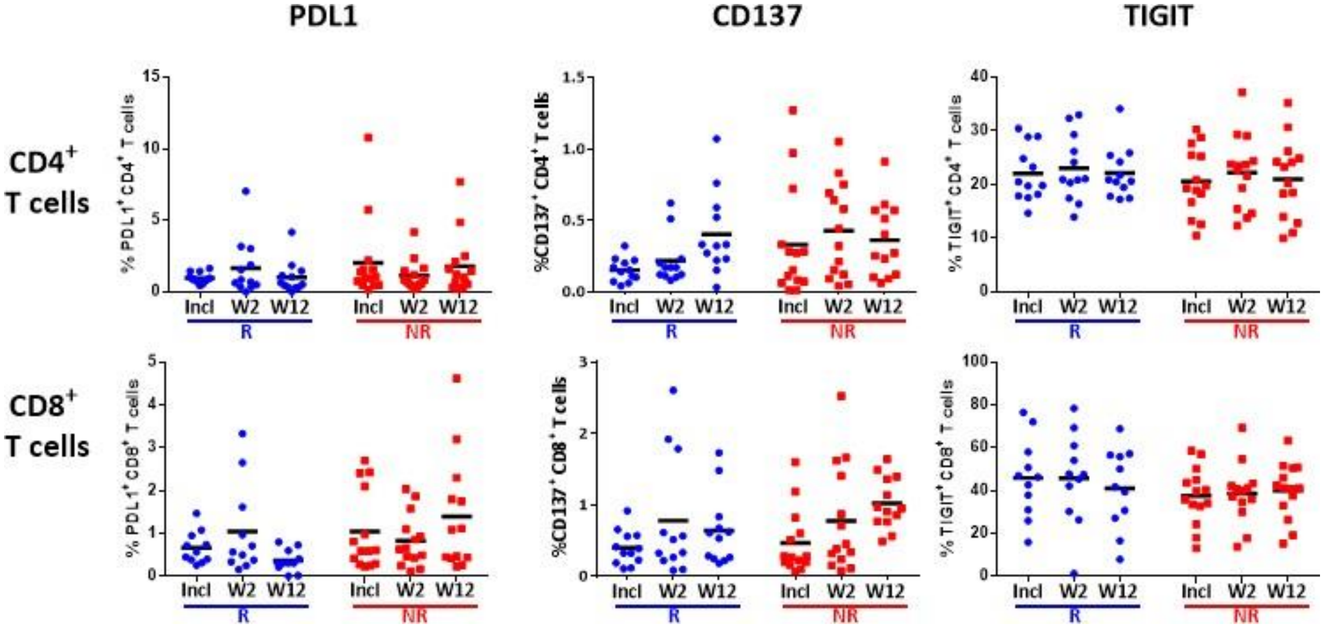

## *Supplementary Figure 4*

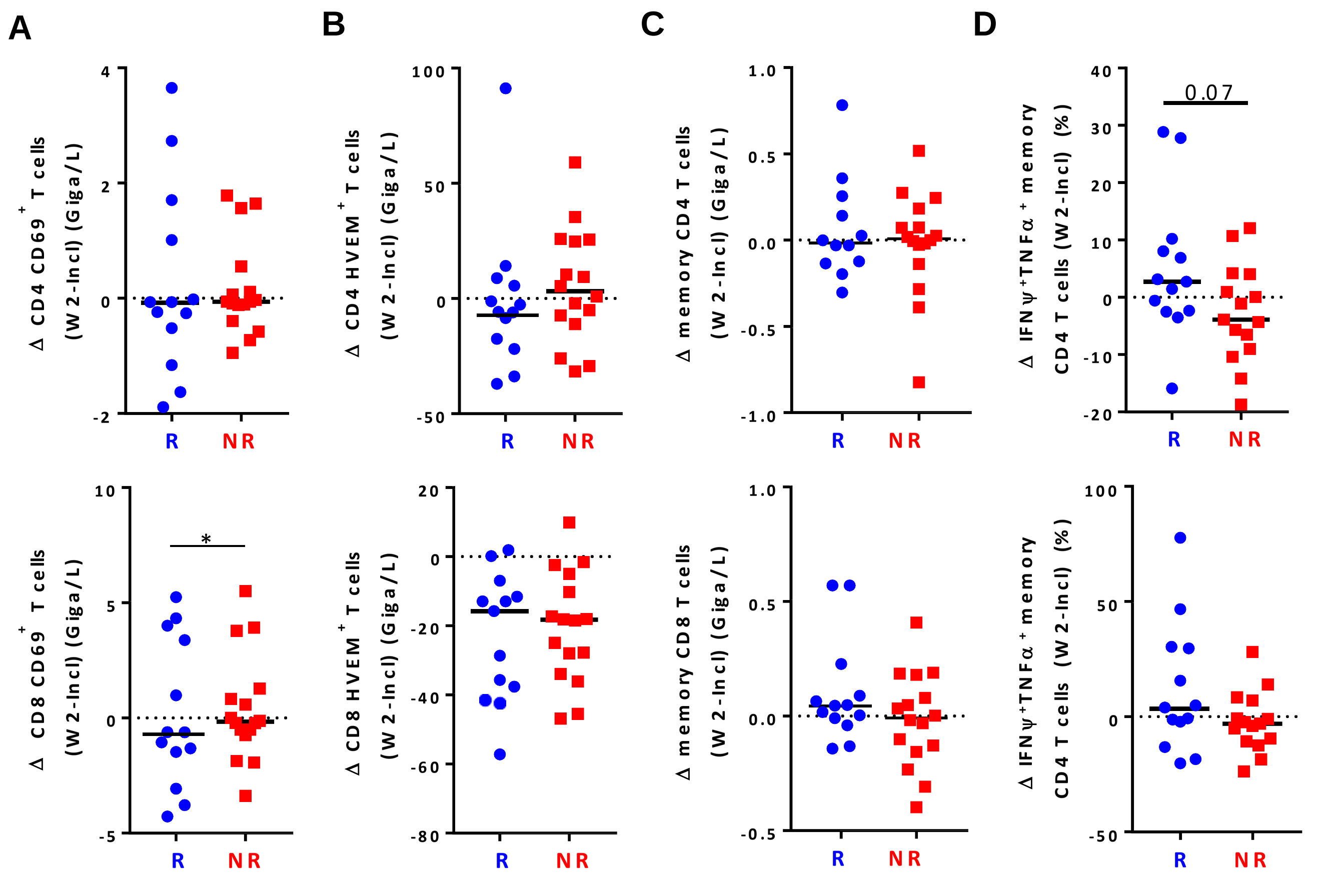

**_Supplementary Figure 5_**

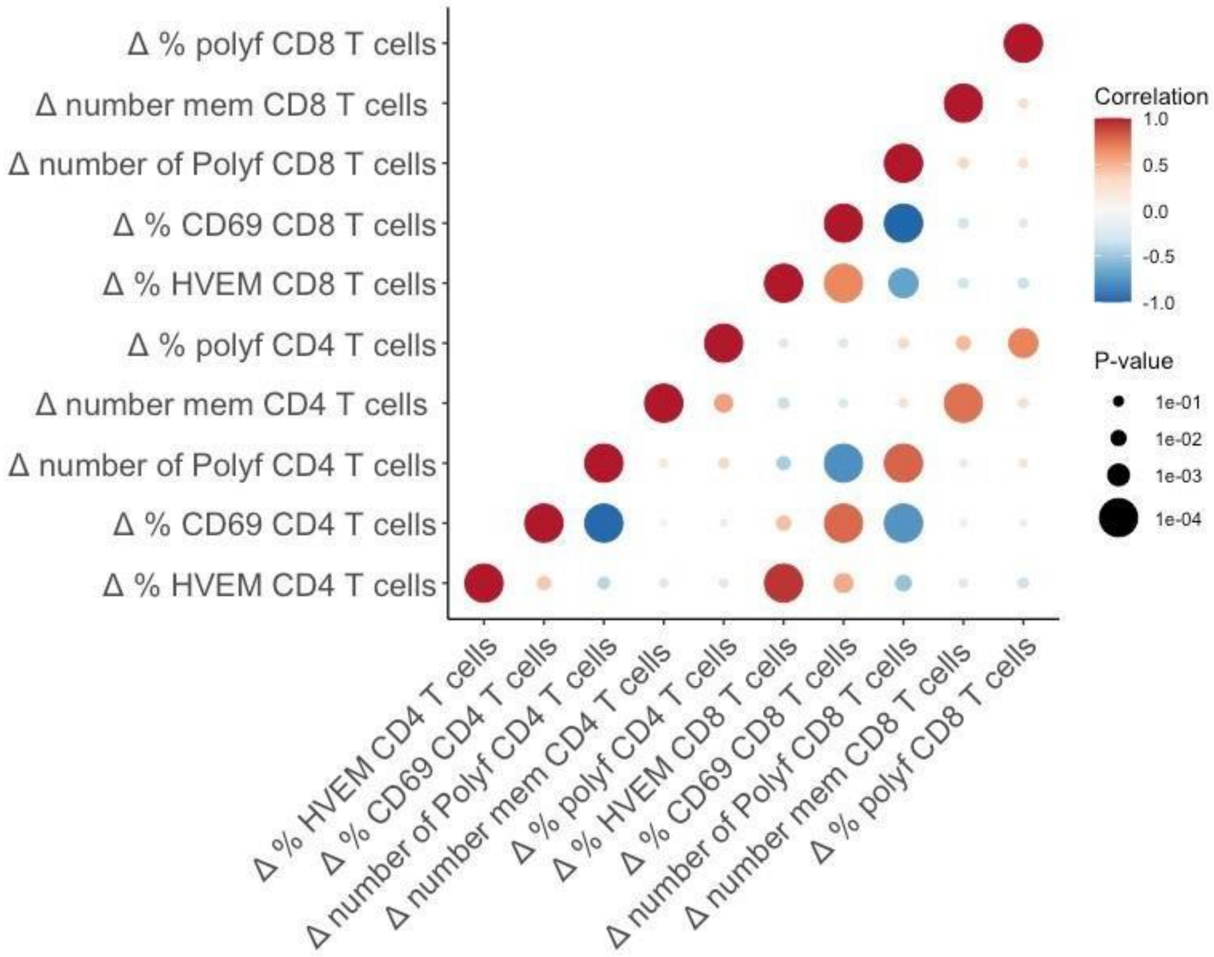

***Supplementary Figure 6***

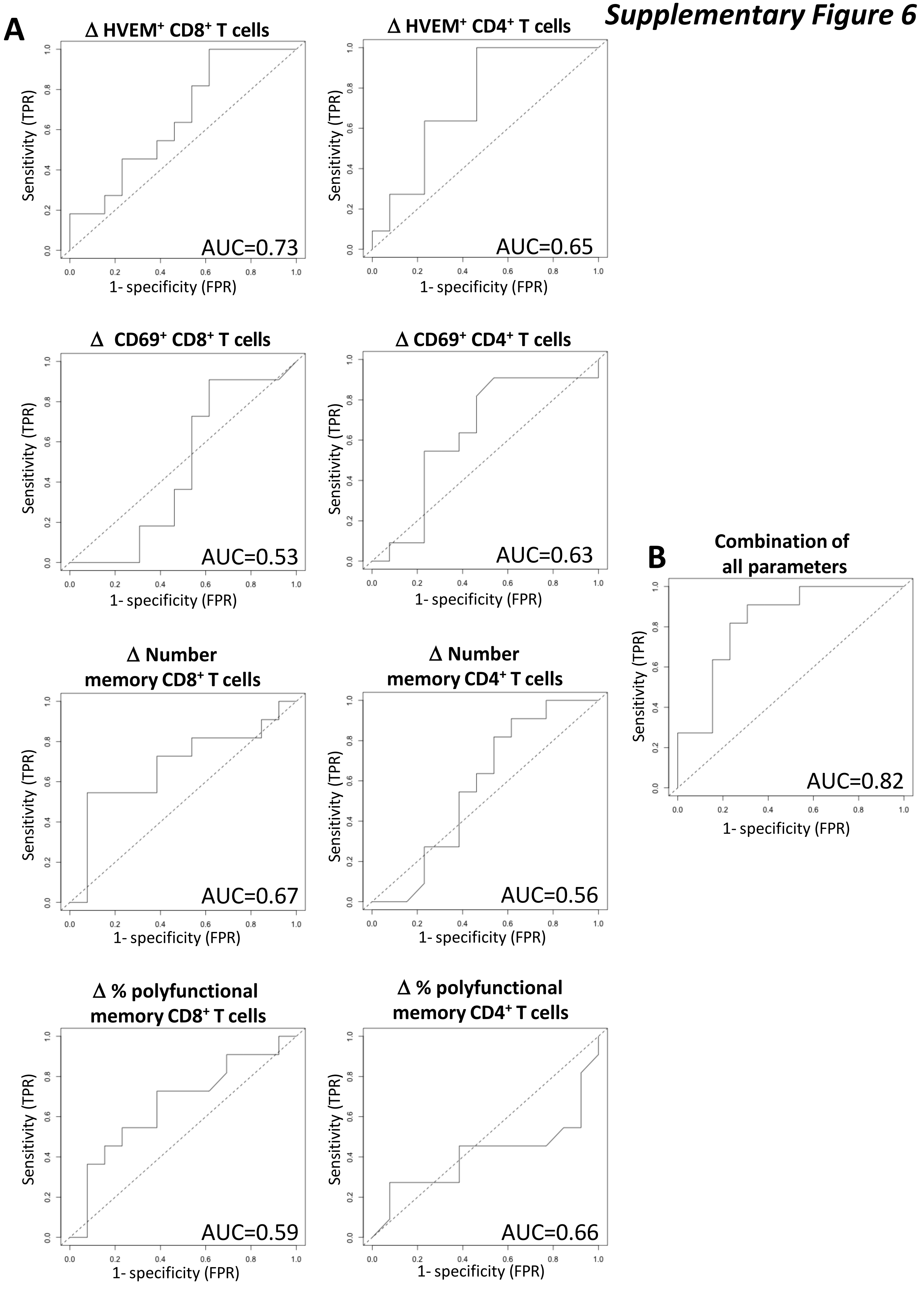

*Supplementary Figure 7*

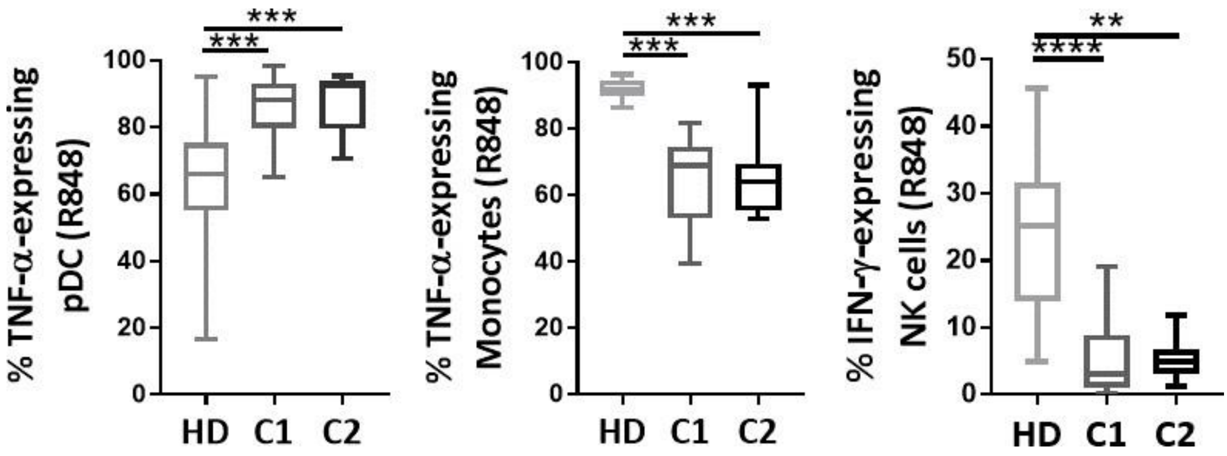

## Supplementary Figure 8

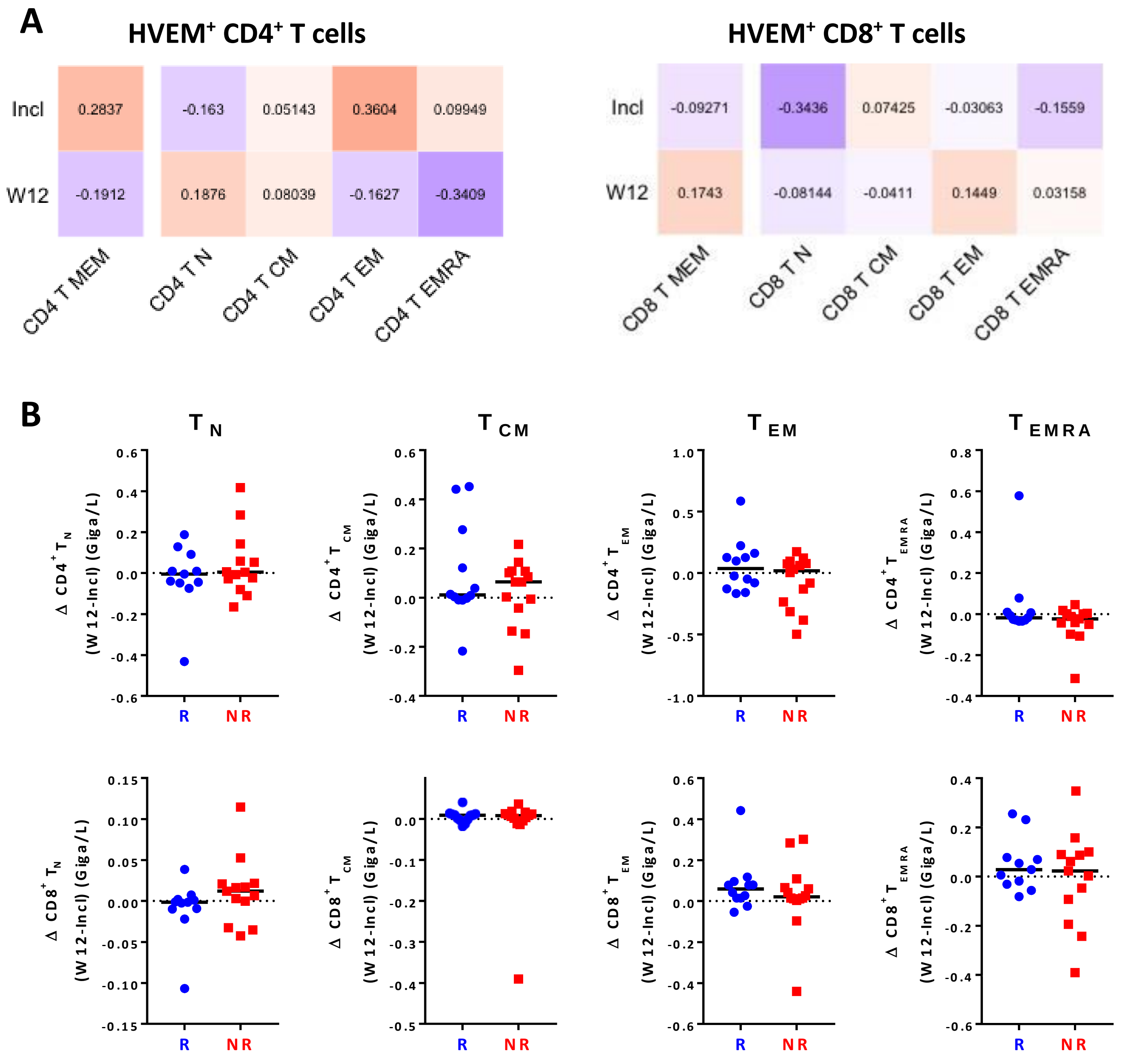

Supplement: Dalle et al supplementary Figures clean.pdf [file KONI_A_2372118_SM9406.pdf]
